# Supplementary material for: Lipemia and its associations with liver disease and dyslipidemia: a cross-sectional study
Source: Lipids Health Dis. 2025 Dec 27;25:25. doi: 10.1186/s12944-025-02845-7 (PMC12853990; doi:10.1186/s12944-025-02845-7)
Supplement: Supplementary file 1 — Supplementary Material 1 [file 12944_2025_2845_MOESM1_ESM.docx]

Supplementary Table 1. Lipemic interference limits for the 27 analytes evaluated in this study

| Analyte | Lipemic Interference Limit |  |
| --- | --- | --- |
|  |  |  |
| TP | Up to 3,000 FTU: No interference |  |
| Alb | Up to 3,000 FTU: No interference |  |
| Urea | Up to 3,000 FTU: No interference |  |
| CREA | Up to 3,000 FTU: No interference |  |
| UA | Up to 3,000 FTU: No interference |  |
| Na | Up to 1,000 mg/dL: No interference |  |
| K | Up to 1,000 mg/dL: No interference |  |
| Cl | Up to 1,000 mg/dL: No interference |  |
| Ca | Up to 2,000 FTU: No interference |  |
| AST | Up to 1,560 FTU: No interference |  |
| ALT | Up to 1,560 FTU: No interference |  |
| AMY | Up to 3,000 FTU: No interference |  |
| TBIL | Up to 5,000 FTU: No interference |  |
| CHE | Up to 3,000 FTU: No interference |  |
| GGT | Up to 3,000 FTU: No interference |  |
| CHOL | Up to 3,000 FTU: No interference |  |
| TG | Not applicable^*^ |  |
| HDL | Up to 3,000 FTU: No interference |  |
| LDL | Up to 3,000 FTU: No interference |  |
| ALP | Up to 1,660 FTU: No interference |  |
| Glc | Up to 1,410 FTU: No interference |  |
| HbA_1c_ | Up to 1,430 FTU: No interference |  |
| WBC | Up to 2,579 FTU: No interference |  |
| RBC | Up to 2,579 FTU: No interference |  |
| Hb | Up to 2,579 FTU: No interference |  |
| Hct | Up to 2,579 FTU: No interference |  |
| Plt | Up to 2,579 FTU: No interference |  |

Data were obtained from the package inserts and manufacturers of the corresponding reagents and analyzers. LIP refers to the lipemic index that is automatically calculated by the TBA™-2000FR analyzer. Formazin turbidity unit (FTU) or intralipid (mg/dL) values were obtained from manufacturer validation studies. These studies used defined turbidity-inducing substances to evaluate lipemic interference. LIP values are not interchangeable with FTU or intralipid (mg/dL) values. Values represent the highest concentration of lipemia expressed either in FTU or intralipid (mg/dL), at which no significant analytical interference was observed. These thresholds reflect manufacturer-reported data under standardized conditions; the actual interference may vary with the analyzer model, reagent lot, and sample matrix. Thus, they should be interpreted as reference thresholds and not as absolute cut-off values.

*Triglycerides (TG) are the principal lipid components of lipoproteins that cause lipemia; therefore, no lipemic interference limit is applicable to TG, and no FTU threshold has been reported by the manufacturer. All instruments used by the reagent manufacturers for lipemic interference testing are listed below.

TP, ALB, CREA, Ca, AST, ALT, TBIL, CHE, GGT, and ALP levels were analyzed using the Hitachi 7180 Clinical Analyzer (Hitachi High-Tech Co., Ltd., Tokyo, Japan). Urea and AMY levels were measured using the LABOSPECT 008 α (Hitachi High-Tech Co., Ltd., Tokyo, Japan). The UA levels were measured using the Hitachi 7170S Clinical Chemistry Analyzer (Hitachi High-Tech Co. Ltd., Tokyo, Japan). Na, K, and Cl were measured using the TBA™-2000FR (Canon Medical Systems Co., Ltd., Otawara, Japan). CHOL, HDL, and LDL levels were measured using LABOSPECT 006 (Hitachi High-Tech Co., Ltd., Tokyo, Japan). Glu was analyzed using the GA09 (A&T Co., Ltd., Yokohama, Japan).

HbA1c was analyzed using the HLC-723® G9 (Tosoh Techno-System Co., Ltd., Tokyo, Japan). The WBC, RBC, Hb, Hct, and Plt were measured using the XE-2100 (Sysmex Co., Ltd., Kobe, Japan).

TP, total protein; Alb, albumin; CREA, creatinine; UA, uric acid; Na, sodium; K, potassium; Cl, chloride; Ca, calcium; AST, aspartate aminotransferase; ALT, alanine aminotransferase; AMY, amylase; TBIL, total bilirubin; CHE, cholinesterase; GGT, gamma-glutamyltransferase; CHOL, total cholesterol; TG, triglycerides; HDL, high-density lipoprotein cholesterol; LDL, low-density lipoprotein cholesterol; ALP, alkaline phosphatase; Glu, glucose; HbA_1c_, glycated hemoglobin; WBC, white blood cells; RBC, red blood cells; Hb, hemoglobin; Hct, hematocrit; Plt, platelets
