# Supplementary material for: Lipemia and its associations with liver disease and dyslipidemia: a cross-sectional study
Source: Lipids Health Dis. 2025 Dec 27;25:25. doi: 10.1186/s12944-025-02845-7 (PMC12853990; doi:10.1186/s12944-025-02845-7)
Supplement: Supplementary file 2 — Supplementary Material 2 [file 12944_2025_2845_MOESM2_ESM.docx]

Supplementary Table 2. Data and sex-stratified statistical analysis for each analyte in the non-, low-, and high-lipemia groups

| Supplementary Table 2-1. | | |  |  |  |  |  |  |
| --- | --- | --- | --- | --- | --- | --- | --- | --- |
| Analyte  (unit) | n | Non-lipemia group (n = 54) | Low-lipemia group (n = 157) | High-lipemia group (n = 147) | *P* | *P* | | |
|  |  |  |  |  |  | Non- vs. low-lipemia group | Non- vs. high-lipemia group | Low- vs. high-lipemia group |
| CREA (μmol/L) | 349 | 80.89 [67.18–91.72] | 79.56 [64.97–96.80] | 84.42 [71.60–103.43] | 0.161 | NA | NA | NA |
| (mg/dL) |  | 0.92 [0.76–1.04] | 0.90 [0.74–1.10] | 0.96 [0.81–1.17] |  |  |  |  |
| UA (μmol/L) | 248 | 0.33 [0.29–0.37] | 0.35 [0.28–0.41] | 0.37 [0.31–0.43] | 0.006 | 0.565 | 0.014* | 0.058 |
| (mg/dL) |  | 5.5 [4.8–6.2] | 5.9 [4.7–6.8] | 6.3 [5.2–7.2] |  |  |  |  |
| ALT  (U/L) | 334 | 22 [16–30] | 23 [17–34] | 25 [18–42] | 0.132 | NA | NA | NA |
| CHE  (U/L) | 194 | 296±83 | 313±100 | 364±95 | <0.001^‡^ | 0.718 | 0.007* | 0.002* |
| GGT  (U/L) | 318 | 32 [21–50] | 36 [23–73] | 42 [27–84] | 0.016 | 0.367 | 0.016* | 0.246 |
| TG (mmol/L) | 166 | 1.42 [1.08–2.18] | 4.22 [2.83–5.85] | 8.42 [6.38–11.31] | <0.001 | <0.001* | <0.001* | <0.001* |
| (mg/dL) |  | 126 [95–193] | 374 [251–518] | 746 [565–1001] |  |  |  |  |
| HDL (mmol/L) | 129 | 1.34 [1.09–1.55] | 1.14 [0.89–1.38] | 0.83 [0.75–1.09] | <0.001 | 0.071 | <0.001* | 0.005* |
| (mg/dL) |  | 52 [42–60] | 44 [34–53] | 32 [29–42] |  |  |  |  |
| RBC (×10^12^/L) | 331 | 4.58 [4.12–4.83] | 4.50 [3.94–4.89] | 4.61 [4.21–4.97] | 0.213 | NA | NA | NA |
| (×10^6^/μL) |  | 4.58 [4.12–4.83] | 4.50 [3.94–4.89] | 4.61 [4.21–4.97] |  |  |  |  |
| Hb (g/L) | 331 | 140 [127–153] | 141 [122–152] | 146 [133–155] | 0.044 | 1.000 | 0.476 | 0.044* |
| (g/dL) |  | 14.0 [12.7–15.3] | 14.1 [12.2–15.2] | 14.6 [13.3–15.5] |  |  |  |  |
| Hct (L/L) | 331 | 0.42 [0.39–0.46] | 0.42 [0.37–0.45] | 0.43 [0.39–0.46] | 0.162 | NA | NA | NA |
| (%) |  | 41.9 [39.3–45.7] | 41.9 [37.2–44.8] | 42.7 [39.4–45.7] |  |  |  |  |
|  |  |  |  |  |  |  |  |  |
| Supplementary Table 2-2. | | |  |  |  |  |  |  |
| Analyte  (unit) | n | Non-lipemia group (n = 54) | Low-lipemia group (n = 89) | High-lipemia group (n = 57) | *P* | *P* | | |
|  |  |  |  |  |  | Non- vs. low-lipemia group | Non- vs. high-lipemia group | Low- vs. high-lipemia group |
| CREA (μmol/L) | 195 | 62.76 [53.92–72.49] | 63.65 [52.16–80.44] | 60.11 [50.83–74.26] | 0.439 | NA | NA | NA |
| (mg/dL) |  | 0.71 [0.61–0.82] | 0.72 [0.59–0.91] | 0.68 [0.58–0.84] |  |  |  |  |
| UA (μmol/L) | 137 | 0.27 [0.23–0.34] | 0.29 [0.23–0.36] | 0.31 [0.26–0.36] | 0.430 | NA | NA | NA |
| (mg/dL) |  | 4.6 [3.9–5.7] | 4.9 [3.9–6.1] | 5.2 [4.3–6.1] |  |  |  |  |
| ALT  (U/L) | 186 | 18 [14–26] | 23 [15–31] | 25 [16–36] | 0.162 | NA | NA | NA |
| CHE  (U/L) | 115 | 315±83 | 334±89 | 379±108 | 0.017^‡^ | 0.621 | 0.016* | 0.090 |
| GGT  (U/L) | 182 | 19 [15–34] | 27 [18–58] | 34 [22–73] | <0.001 | 0.021* | <0.001* | 0.314 |
| TG (mmol/L) | 115 | 1.31 [0.82–1.70] | 4.08 [2.42–5.49] | 7.03 [4.55–9.82] | <0.001 | <0.001* | <0.001* | 0.026* |
| (mg/dL) |  | 116 [73–150] | 361 [215–486] | 622 [403–869] |  |  |  |  |
| HDL (mmol/L) | 84 | 1.53 [1.21–1.82] | 1.33 [1.08–1.99] | 1.12 [0.80–1.42] | 0.017 | 1.000 | 0.018* | 0.092 |
| (mg/dL) |  | 59 [47–71] | 52 [42–77] | 44 [31–55] |  |  |  |  |
| RBC (×10^12^/L) | 180 | 4.24 [3.97–4.52] | 4.22 [3.68–4.45] | 4.19 [3.76–4.78] | 0.584 | NA | NA | NA |
| (×10^6^/μL) |  | 4.24 [3.97–4.52] | 4.22 [3.68–4.45] | 4.19 [3.76–4.78] |  |  |  |  |
| Hb (g/L) | 180 | 128 [119–135] | 129 [109–138] | 130 [116–144] | 0.342 | NA | NA | NA |
| (g/dL) |  | 12.8 [11.9–13.5] | 12.9 [10.9–13.8] | 13.0 [11.6–14.4] |  |  |  |  |
| Hct (L/L) | 181 | 0.39 [0.36–0.42] | 0.39 [0.34–0.42] | 0.39 [0.35–0.43] | 0.395 | NA | NA | NA |
| (%) |  | 38.6 [36.4–41.9] | 38.7 [34.0–41.5] | 39.2 [35.1–43.4] |  |  |  |  |

Data are presented as median [IQR: 25%–75%] or mean ± standard deviation. Both SI and conventional units are presented. Between-group comparisons were performed using the Kruskal–Wallis test with post hoc Dunn–Bonferroni correction or ^‡^one-way analysis of variance with post hoc Tukey’s test. **P* < 0.05, considered statistically significant. Sex stratified data are provided separately in Supplementary Table 2-1 (males) and Supplementary Table 2-2 (females).

CREA, creatinine; UA, uric acid; ALT, alanine aminotransferase; CHE, cholinesterase; GGT, gamma-glutamyl transferase; TG, triglycerides; HDL, high-density lipoprotein cholesterol; RBC, red blood cells; Hb, hemoglobin; Hct, hematocrit.
