# Supplementary material for: Lipemia and its associations with liver disease and dyslipidemia: a cross-sectional study
Source: Lipids Health Dis. 2025 Dec 27;25:25. doi: 10.1186/s12944-025-02845-7 (PMC12853990; doi:10.1186/s12944-025-02845-7)
Supplement: Supplementary file 3 — Supplementary Material 3 [file 12944_2025_2845_MOESM3_ESM.docx]

Supplementary Table 3. Reference ranges for the 27 analytes evaluated in this study

| Analyte  (unit) | Reference Range, male | Reference Range, female |  |
| --- | --- | --- | --- |
|  |  |  |  |
| TP  (g/L) | 66–81 | 66–81 |  |
| (g/dL) | 6.6–8.1 | 6.6–8.1 |  |
| Alb (g/L) | 41–52 | 41–52 |  |
| (g/dL) | 4.1–5.2 | 4.1–5.2 |  |
| Urea (mmol/L) | 2.7–7.1 | 2.7–7.1 |  |
| (mg/dL) | 8–20 | 8–20 |  |
| CREA (μmol/L) | 58–94 | 41–70 |  |
| (mg/dL) | 0.65–1.07 | 0.46–0.79 |  |
| UA (μmol/L) | 220–463 | 152–328 |  |
| (mg/dL) | 3.7–7.8 | 2.6–5.5 |  |
| Na  (mmol/L) | 138–145 | 138–145 |  |
| K  (mmol/L) | 3.6–4.8 | 3.6–4.8 |  |
| Cl  (mmol/L) | 101–108 | 101–108 |  |
| Ca  (mmol/L) | 2.18–2.53 | 2.18–2.53 |  |
| (mg/dL) | 8.8–10.1 | 8.8–10.1 |  |
| AST (U/L) | 13–30 | 13–30 |  |
| ALT  (U/L) | 10–42 | 7–23 |  |
| AMY  (U/L) | 44–132 | 44–132 |  |
| TBIL (μmol/L) | 6.8–26.3 | 6.8–26.3 |  |
| (mg/dL) | 0.4–1.5 | 0.4–1.5 |  |
| CHE  (U/L) | 240–486 | 201–421 |  |
| GGT  (U/L) | 13–64 | 9–32 |  |
| CHOL (mmol/L) | 3.7–6.4 | 3.7–6.4 |  |
| (mg/dL) | 142–248 | 142–248 |  |
| TG (mmol/L) | 0.5–2.6 | 0.3–1.3 |  |
| (mg/dL) | 40–234 | 30–117 |  |
| HDL (mmol/L) | 1.0–2.3 | 1.2–2.7 |  |
| (mg/dL) | 38–90 | 48–103 |  |
| LDL (mmol/L) | 1.7–4.2 | 1.7–4.2 |  |
| (mg/dL) | 65–163 | 65–163 |  |
| ALP (U/L) | 38–113 | 38–113 |  |
| Glu  (mmol/L) | 4.1–6.1 | 4.1–6.1 |  |
| (mg/dL) | 73–109 | 73–109 |  |
| HbA_1c_ (mmol/mol) | 30–42 | 30–42 |  |
| (%) | 4.9–6.0 | 4.9–6.0 |  |
| WBC  (×10^9^/L) | 3.3–8.6 | 3.3–8.6 |  |
| (×10^3^/μL) | 3.3–8.6 | 3.3–8.6 |  |
| RBC (×10^12^/L) | 4.35–5.55 | 3.86–4.92 |  |
| (×10^6^/μL) | 4.35–5.55 | 3.86–4.92 |  |
| Hb (g/L) | 137–168 | 116–148 |  |
| (g/dL) | 13.7–16.8 | 11.6–14.8 |  |
| Hct (L/L) | 0.41–0.50 | 0.35–0.44 |  |
| (%) | 40.7–50.1 | 35.1–44.4 |  |
| Plt (×10^9^/L) | 158–348 | 158–348 |  |
| (×10^3^/μL) | 158–348 | 158–348 |  |

This supplementary table presents the reference ranges for the 27 analytes based on the common reference intervals published by the Japanese Committee for Clinical Laboratory Standards Japanese Shared Reference Intervals (2022 edition) [31]. For analytes with sex-specific differences, separate ranges were provided for males and females, whereas for analytes without reported sex-specific differences, identical values were obtained for both sexes.

TP, total protein; Alb, albumin; CREA, creatinine; UA, uric acid; Na, sodium; K, potassium; Cl, chloride; Ca, calcium; AST, aspartate aminotransferase; ALT, alanine aminotransferase; AMY, amylase; TBIL, total bilirubin; CHE, cholinesterase; GGT, gamma-glutamyltransferase; CHOL, total cholesterol; TG, triglycerides; HDL, high-density lipoprotein cholesterol; LDL, low-density lipoprotein cholesterol; ALP, alkaline phosphatase; Glu, glucose; HbA_1c_, glycated hemoglobin; WBC, white blood cells; RBC, red blood cells; Hb, hemoglobin; Hct, hematocrit; Plt, platelets
