# Supplementary material for: Lipemia and its associations with liver disease and dyslipidemia: a cross-sectional study
Source: Lipids Health Dis. 2025 Dec 27;25:25. doi: 10.1186/s12944-025-02845-7 (PMC12853990; doi:10.1186/s12944-025-02845-7)
Supplement: Supplementary file 4 — Supplementary Material 4 [file 12944_2025_2845_MOESM4_ESM.pdf]

Revised manuscript (1203)_Mami Osawa.docx

Niigata University

Document Details

Submission ID

trn:oid:::3117:535726404

Submission Date

Dec 3, 2025, 4:06 PM GMT+9

Download Date

Dec 3, 2025, 4:11 PM GMT+9

File Name

Revised manuscript (1203)_Mami Osawa.docx

File Size

298.1 KB

65 Pages

11,173 Words

63,183 Characters

10% Overall Similarity

The combined total of all matches, including overlapping sources, for each database.

Filtered from the Report

- Bibliography
- Small Matches (less than 10 words)

Match Groups

40 Not Cited or Quoted 8%
Matches with neither in-text citation nor quotation marks

11 Missing Quotations 2%
Matches that are still very similar to source material

0 Missing Citation 0%
Matches that have quotation marks, but no in-text citation

0 Cited and Quoted 0%
Matches with in-text citation present, but no quotation marks

Top Sources

9% Internet sources
7% Publications
0% Submitted works (Student Papers)

Integrity Flags

0 Integrity Flags for Review

No suspicious text manipulations found.

Our system's algorithms look deeply at a document for any inconsistencies that would set it apart from a normal submission. If we notice something strange, we flag it for you to review.

A Flag is not necessarily an indicator of a problem. However, we'd recommend you focus your attention there for further review.

Match Groups

- **40** Not Cited or Quoted 8%
  Matches with neither in-text citation nor quotation marks
- **11** Missing Quotations 2%
  Matches that are still very similar to source material
- **0** Missing Citation 0%
  Matches that have quotation marks, but no in-text citation
- **0** Cited and Quoted 0%
  Matches with in-text citation present, but no quotation marks

Top Sources

9% Internet sources
7% Publications
0% Submitted works (Student Papers)

Top Sources

The sources with the highest number of matches within the submission. Overlapping sources will not be displayed.

#	Type	Source	Match
1	Internet	www.mdpi.com	1%
2	Internet	doaj.org	<1%
3	Internet	www.frontiersin.org	<1%
4	Internet	pmc.ncbi.nlm.nih.gov	<1%
5	Internet	www.life-science-alliance.org	<1%
6	Internet	public-pages-files-2025.frontiersin.org	<1%
7	Internet	research.vu.nl	<1%
8	Internet	www.researchsquare.com	<1%
9	Internet	bmcmusculoskeletdisord.biomedcentral.com	<1%
10	Internet	www.explorationpub.com	<1%

11 Internet
www.nature.com <1%

12 Internet
assets-eu.researchsquare.com <1%

13 Internet
bmcpalliatcare.biomedcentral.com <1%

14 Internet
iperfusion.org <1%

15 Publication
Kimi Kurotobi, Akiko Yamamoto, Akemi Kikuta, Takao Hanawa. "Short term evalu... <1%

16 Publication
Masahiro Takahashi, Namiko Uchino. "Risk factors of hypermagnesemia in end-st... <1%

17 Publication
Tetsuya Matoba, Shunsuke Katsuki, Yasuhiro Nakano, Takuro Kawahara et al. "Eff... <1%

18 Internet
df6sxcketz7bb.cloudfront.net <1%

19 Publication
Muratsubaki H.. "Hypertriglyceridemia Associated with Decreased Post-Heparin P... <1%

20 Internet
www.japscjournal.com <1%

21 Internet
ousar.lib.okayama-u.ac.jp <1%

22 Internet
wprim.whocc.org.cn <1%

23 Publication
Gabriel M. Sousa, Rodrigo C. Oliveira, Mariana M. Pereira, Raymundo Paraná, Mar... <1%

24 Internet
bmccardiovascdisord.biomedcentral.com <1%

25	Internet	
osp.mans.edu.eg		<1%
26	Publication	
Yoshihiro Matsumoto, Yasuo Mori, Shinji Kageyama, Kazuo Arihara et al. "Change...		<1%
27	Internet	
archive.org		<1%
28	Internet	
assets.researchsquare.com		<1%
29	Internet	
bmcnutr.biomedcentral.com		<1%
30	Internet	
d.docksci.com		<1%
31	Internet	
journals.lww.com		<1%
32	Internet	
www.icdremediator.com		<1%
33	Internet	
www.tdx.cat		<1%
34	Publication	
Namki Hong. "High Fatty Liver Index and Fracture Risk: Clinical Implications", Gut...		<1%
35	Internet	
medicalresearch.med.bg.ac.rs		<1%
36	Internet	
repositorio.uam.es		<1%
37	Internet	
repository.niddk.nih.gov		<1%
38	Internet	
www.jstage.jst.go.jp		<1%

39 Internet

www.science.gov **<1%**

Lipemia and its Associations with Liver Disease and Dyslipidemia: A Cross-sectional Study

Mami Osawa[a,]*, Yasunobu Matsuda[a], Takashi Ushiki[b], Toshifumi Wakai[c]

[a]Department of Medical Technology Science, Niigata University Graduate School of Health Sciences, 2-746 Asahimachi-dori, Chuo-Ku, Niigata 951-8518, Japan

[b]Division of Hematology and Oncology, Niigata University Graduate School of Health Sciences, 2-746 Asahimachi-dori, Chuo-Ku, Niigata 951-9518, Japan.

[c]Division of Digestive and General Surgery, Niigata University Graduate School of Medical and Dental Sciences, 2-746 Asahimachi-dori, Chuo-Ku, Niigata 951-8518, Japan

***Corresponding author:** Mami Osawa, PhD

Department of Medical Technology Science, Niigata University Graduate School of Health Sciences, 2-746 Asahimachi-dori, Chuo-Ku, Niigata 951-8518, Japan

E-mail: oswmami@clg.niigata-u.ac.jp

Abstract

Background: Lipemia is characterized by a milky appearance of plasma, which can be easily detected using an automated clinical chemistry analyzer. To date, few studies have evaluated the relationship between lipemia values and clinical test data other than lipid parameters. This study aimed to analyze the relationship among lipemia, clinical test data, and associated disorders.

Methods: This cross-sectional study examined 730 specimens from patients with and without lipemia who visited the Niigata University Medical and Dental Hospital in Japan. The participants were divided according to their lipemia index (LIP) into non- (<1.5 LIP), low- (1.5–4.9 LIP), and high-lipemia (≥5.0 LIP) groups. Twenty-seven clinical analytes were analyzed, and their associations with the extent of lipemia were investigated using group comparisons, multinomial logistic regression, and correlation analyses. The prevalence of dyslipidemia and liver disease was also evaluated in the lipemic group.

Results: The lipemic group exhibited higher total cholesterol and triglyceride levels than the non-lipemia group ($P < 0.01$). The high-lipemia group demonstrated significantly higher median liver chemistries than the non-lipemia group: aspartate aminotransferase, 27 U/L (interquartile range [IQR], 22–35 U/L) vs. 23 U/L (IQR, 20–28 U/L); gamma-glutamyltransferase, 39 U/L (IQR, 26–79 U/L) vs. 24 U/L (IQR, 16–40 U/L) ($P < 0.01$). Individuals in the high-lipemia group had a higher complication rate of concomitant

dyslipidemia and liver disease.

Conclusion: Lipemia was associated with elevated lipid metabolism-related parameters and liver chemistries. The LIP can be used to evaluate risks associated with liver disease and dyslipidemia.

Keywords

Lipemia, Liver Diseases, Dyslipidemias, Biomarkers, Cross-Sectional Studies

Background

Lipemia is marked by elevated levels of large lipoproteins (≥35 nm), including chylomicrons (CM) and very low-density lipoprotein (VLDL), resulting in a hazy, milky appearance of the plasma [1, 2]. CM are triglyceride (TG)-rich lipoproteins produced in the intestine following ingestion of dietary fat, with particle sizes varying from 70 to 1000 nm with individual differences [3, 4]. VLDL is produced by the liver and is classified according to its size as small (27–35 nm), medium (35–60 nm), or large (60–200 nm) [1]. VLDL sizes correlate well with the extent of lipemia, and higher VLDL sizes and concentrations have been reported as the main causes of lipemia in conditions of insulin-resistance [1].

Lipemia is usually caused by excessive consumption of dietary fat and large amounts of glucose and alcohol [5–8]. Moreover, certain medical reagents such as diuretics, human immunodeficiency virus (HIV) protease inhibitors, beta-blockers, selective serotonin reuptake inhibitors, estrogens, glucocorticoids, and intravenous lipid emulsion administration have also been implicated in lipemia [4, 9–12]. The incidence of lipemia is low but not rare. Tian et al. reported that 0.5% of 10,000 serum samples were lipemic, and the frequency was higher in inpatients than in outpatients at medical facilities [13].

Various pathological disorders and postprandial conditions are closely associated with lipemia. Lipemia is primarily caused by a spectrum of lipid metabolic disorders, including familial hyperlipidemia types I, IV, and V (World Health Organization phenotype) and familial

lipoprotein lipase (LPL) deficiency [10, 14]. It is also a secondary pathological factor in non-lipidemic disorders, including diabetes mellitus, pancreatitis, alcoholism, kidney disease, hypothyroidism, non-alcoholic fatty liver disease, and HIV infection [4, 10, 11]. Of note, it has been widely recognized that liver disease and metabolic dysfunction are important underlying factors contributing to lipemia. Metabolic dysfunction-associated steatotic liver disease (MASLD) has emerged as the most common type of chronic liver disease globally, affecting up to 30% of the adults in developed countries. The Fatty Liver Index (FLI) has been validated as an accurate, non-invasive screening tool for hepatic steatosis, incorporating four readily available parameters: serum TGs, the body mass index, gamma-glutamyl transferase (GGT), and waist circumference [15, 16]. The FLI demonstrates excellent diagnostic performance for identifying hepatic steatosis compared with that of imaging gold standards. In this study, it was hypothesized that the Lipemia Index (LIP), a quantitative measure of sample turbidity reflecting circulating chylomicrons, VLDL, and other TG-rich lipoproteins, might exhibit measurable associations with a wide range of routine laboratory parameters, including hematological, glucose-related, and biochemical tests.

Although the mechanism of lipemia in such diseases remains unclear, studies have suggested that it is caused by the reduced or impaired catabolism of LPL. LPL is the key enzyme that limits the rate of TG degradation in lipoproteins, such as CM and VLDL [17]. For example, in insulin-resistance syndrome and diabetes, reduced LPL activity results in impaired VLDL

clearance and accelerated production of large VLDL particles [1, 18]. In some patients with uremia, the ratio of the LPL activator apolipoprotein C-II/LPL inhibitor apolipoprotein C-III decreases [19], which may lead to impaired LPL activity.

Based on the above evidence, an investigation was conducted to determine whether analyzing the lipemic condition of the plasma or serum might be useful for screening individuals with pathogenic disorders. Currently, most clinical chemistry analyzers are equipped with automated measurement systems to detect interference from lipemia, and the extent of lipemia can be rapidly determined using lipemic indices. To date, only a few studies have examined the possibility of disease screening using the LIP of serum samples [10, 20–23]. In this study, a retrospective analysis was performed on clinical test data from both non-lipemic and lipemic plasma samples to investigate whether an association exists between the extent of lipemia, clinical tests, and the prevalence of disorders. This study represents one of the first systematic evaluation of lipemia as a potential dual biomarker for liver disease and dyslipidemia through comprehensive multiparameter analysis in a cross-sectional study. This study addresses a significant gap in understanding the interrelated pathophysiology between lipemic samples and diverse clinical conditions.

Methods

Participants

iThenticate Page 12 of 71 - Integrity Submission Submission ID trn:oid:::3117:535726404

A cross-sectional study was performed using lipemic plasma samples from 730 individuals visiting the Niigata University Medical and Dental Hospital in Japan, a tertiary-care institution that not only specializes in metabolic, endocrine, gastrointestinal, and related diseases but also provides comprehensive care for a broad range of common conditions. The study was conducted over 15 months, from January 2021 to March 2022. This study included 622 patients with lipemia and 108 non-lipemic controls; the latter were randomly selected to serve as the control group. The participants were aged between 18 and 96 years. Pregnant patients, as well as those with cytomegalovirus or HIV infection, multiple myeloma, monoclonal gammopathy, or paraproteinemia were excluded [24]. Specimens other than blood, plasma, such as pleural effusion or ascites were excluded from the study. Of the 622 lipemic cases, 172 exhibited quantitative LIP values required for correlation analysis. The remaining 558 (450 lipemic with qualitative LIP only and 108 non-lipemic controls) patients were included in the other analyses presented in the tables and figure.

Data collection and laboratory procedures

To investigate the relationship between lipemia and the clinical laboratory data, 27 biochemical analytes were analyzed. The clinical chemistry automated analyzer TBA™-2000FR (Canon Medical Systems Co., Ltd., Otawara, Japan) was used to measure total protein (TP), albumin (Alb), urea, creatinine (CREA), uric acid (UA), sodium (Na), potassium (K), chlorine (Cl),

calcium (Ca), aspartate aminotransferase (AST), alanine aminotransferase (ALT), amylase (AMY), total bilirubin (TBIL), cholinesterase (CHE), GGT, total cholesterol (CHOL), TG, high-density lipoprotein cholesterol (HDL), low-density lipoprotein cholesterol (LDL), and alkaline phosphatase (ALP) levels. Glucose (Glu) and glycated hemoglobin (HbA_{1c}) were analyzed using the glucose analyzer GA09 (A&T Co., Ltd., Yokohama, Japan) and the automatic glycated hemoglobin analyzer HLC-723® G9 (Tosoh Techno-System Co., Ltd., Tokyo, Japan), respectively. An automatic hematology analyzer XE-5000 (Sysmex Co., Ltd., Kobe, Japan) was used to assess the white blood cell (WBC), red blood cell (RBC), hemoglobin (Hb), hematocrit (Hct), and platelet (Plt) counts. Abnormal values were assigned to instances in which laboratory parameters deviated from established reference ranges.

At this institution, blood samples are generally recommended to be obtained after overnight fasting. Because this study was retrospective, it was not possible to verify that all samples were strictly collected after fasting; however, it was confirmed that blood glucose samples were generally obtained under fasting conditions.

In this study, plasma samples were processed in accordance with standard laboratory protocols, but without undergoing lipid-clearing procedures such as ultracentrifugation or lipid extraction. This was done intentionally to preserve the samples in as natural a state as possible to allow automatic measurement of the LIP by the analyzer and to ensure that follow-up or additional laboratory tests, which are frequently requested at the present institution, could be

performed without compromising sample integrity. It is widely accepted that lipemia can interfere with the results of clinical chemistry tests [25–28]. Therefore, for items with suspected absorbance errors or where confirmation against previous values is necessary, the measurement values obtained after sample dilution are provided. If the measurement value remained unstable even after dilution, it was reported as "unmeasurable or for reference only." The details of the lipemic interference for each analyte are provided in Supplementary Table 1. The formazin turbidity unit (FTU) or intralipid (mg/dL) values in this table were derived from lipemic interference validation studies performed by the reagent manufacturers. The LIP values generated by the TBA™-2000FR analyzer are derived from multi-wavelength absorbance measurements and cannot be directly compared with FTU or intralipid (mg/dL) values reported in manufacturer-performed lipemic-interference validation studies. The specific analyzers used in the validation studies are listed in the footnote of Supplementary Table 1. Lipemic properties of the samples were detected using a clinical chemistry automated analyzer (TBA™-2000FR), and the extent of lipemia was expressed as LIP using the combination of measuring lights at multiple wavelengths of 500/524, 572/604, 628/660, and 524/804 nm according to the manufacturer's specifications. In this analyzer, 1 LIP unit corresponds to the turbidity produced by a 0.5% solution of INTRAFAT®, an intravenous fat emulsion containing soybean oil (Takeda Pharmaceutical Co., Ltd., Osaka, Japan). According to the specifications of the LIP analyzer, the extent of lipemia was expressed as non-lipemic (<1.5 LIP), +1 (1.5–3.4 LIP), +2

iThenticate Page 15 of 71 - Integrity Submission Submission ID trn:oid:::3117:535726404

(3.5–4.9 LIP), +3 (5.0–5.9 LIP), and +4 (≥6.0 LIP). Participants were classified into three groups based on their LIP values: non-lipemia (<1.5 LIP), low-lipemia (1.5–4.9 LIP), and high-lipemia (≥5.0 LIP) groups.

Dyslipidemia was defined by physicians as meeting any one of the diagnostic criteria set out in the Japan Atherosclerosis Society (JAS) Guidelines for the Prevention of Atherosclerotic Cardiovascular Diseases 2022 [29]. According to these guidelines, the diagnostic thresholds for dyslipidemia applied in the present study were LDL ≥3.62 mmol/L, HDL <1.03 mmol/L, and/or TG ≥1.69 mmol/L. Fasting and non-fasting samples were used for the assessment. However, in cases where the individual was not fasting, TG levels had to be ≥1.98 mmol/L. Patients meeting any of these criteria or with a documented history of dyslipidemia were classified as having dyslipidemia.

Liver disease was defined by physicians as meeting any of the following criteria: (1) persistent elevated liver enzymes for more than 6 months; (2) imaging-confirmed hepatic abnormalities (e.g. hepatic steatosis, cirrhosis or other parenchymal disease) on an abdominal ultrasound, computed tomography (CT), or magnetic resonance image (MRI) scans; (3) positive viral hepatitis markers (e.g. HBsAg or HCV antibody positivity); (4) liver biopsies; and (5) a documented history of a liver disease diagnosis [30]. Patients meeting any of these criteria were classified as having liver disease and identified using International Classification of Diseases, 10th Revision (ICD-10) codes.

Statistical analyses

Normality was assessed using the Shapiro–Wilk test. For variables with a normal distribution, a one-way analysis of variance (ANOVA) was performed, and the results were expressed as means with standard deviations. Non-normally distributed variables were analyzed using the Kruskal–Wallis test, with the results expressed as medians (interquartile range [IQR]). Categorical variables were evaluated using the chi-squared test and reported as frequencies (percentages). To analyze the relationship between lipemia and each variable using multinomial logistic regression, multinomial logistic regression analysis was performed after adjusting for age and sex. Spearman's rank correlation coefficient was used to evaluate the correlations between LIP and several parameters. Abnormal values were defined based on common reference intervals established by the Japanese Committee for Clinical Laboratory Standards Japanese Shared Reference Intervals (2022 edition) [31]. Values above the reference range were considered "abnormal". Statistical significance was set at $P < 0.05$. Statistical analyses were performed using IBM SPSS Statistics v.28.0.1 (IBM Corp., Armonk, NY, USA). Missing data were addressed on a variable-by-variable basis instead of excluding all patients. Only records with available data for each laboratory parameter were included in the analysis, whereas all other available results from the same patients were retained. The number of participants included in each analysis is listed in the corresponding table. Logistic regression analyses were

conducted separately for each laboratory variable, using a multinomial model in which the dependent variable was lipemia category (non, low, or high) and the independent variables were age and sex. This approach maximizes data use, minimizes unnecessary exclusions, and prevents multicollinearity.

Results

Clinical characteristics

The clinical characteristics and laboratory data of the non-, low-, and high-lipemia groups are presented in Table 1. Patient age was significantly associated with the lipemic status. The median ages were 68 [IQR, 55–75], 59 [IQR, 49–68], and 54 [IQR, 46–65] years in the non-, low-, and high-lipemia groups, respectively (P = 0.001; non- vs. high-lipemia groups, P < 0.001). The proportion of male patients significantly increased according to the extent of lipemia, from 50.0% in the non-lipemia group to 72.1% in the high-lipemia group (P < 0.001).

[Insert Table 1 here]

Clinical chemistry and blood count data

Statistical analysis of 27 laboratory parameters revealed significant differences in lipid and non-lipid metabolic parameters between the non-lipemic and lipemic groups (Table 1, Supplementary Table 2). The manufacturer-determined lipemic interference limits for each

analyte are summarized in Supplementary Table 1. This table supports Table 1 by indicating the lipemia levels that did not affect the test results. As for lipid metabolic parameters, post-hoc tests revealed a significant reduction in HDL and a notable increase in CHOL and TG levels in both the low- and high-lipemia groups compared with those of the non-lipemia group ($P < 0.05$) (Table 1). Significant increases and decreases in TG and HDL levels were observed in the low- and high-lipemia groups ($P < 0.001$ and $P < 0.001$, respectively) (Table 1). For non-lipid metabolic parameters, both the low- and high-lipemia groups showed significant increases in GGT, ALP, WBC, and Plt values, and a significant decrease in TBIL levels compared with those of the non-lipemia group ($P < 0.05$) (Table 1). In addition, the high-lipemia group exhibited significant elevations in CREA, UA, AST, ALT, CHE, and Hb levels compared with those of the non-lipemia group ($P < 0.05$) (Table 1). The UA, CHE, GGT, and Hb levels were significantly elevated in the high-lipemia group compared with those of the low-lipemia group ($P < 0.05$) (Table 1). The median fasting blood glucose level in the non-, low- and high-lipemia groups was 6.61 [IQR, 5.66–7.33], 6.33 [IQR, 5.75–8.72], and 6.52 [IQR, 5.50–7.58] mmol/L, whereas the levels of Glu and HbA_{1c} did not differ significantly between the low- and high-lipemia groups and the non-lipemia group (Table 1). To examine the variables influenced by the extent of lipemia, the relationship between clinical data and lipemia groups was further analyzed using multinomial logistic regression analysis (Table 2). Before the analysis, each variable was adjusted for age and sex. Seven variables (Urea, AST, CHOL, TG, ALP, WBC,

and Plt) demonstrated significant odds ratios in both the low- and high-lipemia groups compared with the non-lipidemic group (Table 2). A significant increase was found in UA, Ca, CHE, and GGT odds ratios and significantly reduced Cl and HDL levels in the high-lipemia group compared with those in the non-lipemia group (Table 2).

Based on these results, this study investigated the association between lipemia and laboratory values for liver chemistries above the reference intervals. The percentages of specimens with AST levels above the reference interval were 19.2%, 30.0%, and 37.2% in the non-, low-, and high-lipemia groups, respectively (non- vs. low-lipemia groups, P = 0.042; non- vs. high-lipemia groups, P = 0.002) (Table 3). The percentages of specimens with GGT levels above the reference interval were 16.3%, 29.8%, and 32.0% for male participants in the non-, low-, and high-lipemia groups, respectively, and 27.1%, 43.2%, and 50.9% for female participants in the non-, low-, and high-lipemia groups, respectively (male participants: non- vs. low-lipemia group, P = 0.065; non- vs. high-lipemia group, P = 0.037; female participants: non- vs. low-lipemia group, P = 0.067; non- vs. high-lipemia group, P = 0.014) (Table 3, Supplementary Table 3).

[Insert Table 2 here]

Table 3. Proportion of liver chemistries values above the reference interval

Values above the reference interval	Non-lipemia group (n = 99)	Low-lipemia group (n = 230)	High-lipemia group (n = 191)	*P*-value	
				Non- vs. low-lipemia group	Non- vs. high-lipemia group
AST	19 (19.2)	69 (30.0)	71 (37.2)	0.042*	0.002*
	Non-lipemia group (n = 49)	Low-lipemia group (n = 141)	High-lipemia group (n = 128)	*P*-value	
				Non- vs. low-lipemia group	Non- vs. high-lipemia group
GGT, male	8 (16.3)	42 (29.8)	41 (32.0)	0.065	0.037*
	Non-lipemia group (n = 48)	Low-lipemia group (n = 81)	High-lipemia group (n = 53)	*P*-value	
				Non- vs. low-lipemia group	Non- vs. high-lipemia group
GGT, female	13 (27.1)	35 (43.2)	27 (50.9)	0.067	0.014*

Data are expressed as n (%). GGT was presented as a sex-specific result as reference ranges differ between males and females.

Statistical analyses were performed using the chi-square test. *Statistically significant.

AST, aspartate aminotransferase; GGT, gamma-glutamyl transferase

To evaluate the clinical utility as a laboratory data indicator, the correlation coefficients between LIP and lipid metabolic parameters (CHOL, TG, and HDL) and liver chemistries (AST and GGT) were analyzed using Spearman's rank correlation coefficients (Table 4). Based on

the collected data, a positive correlation was observed between TG and LIP (LIP vs. TG, $r = 0.563$; $P < 0.001$) (Table 4). The relationship between liver chemistries and LIP was not quantitatively significant (Table 4) but was relatively significant (Table 2).

Table 4. Correlation between the lipemia index and the five analytes

**Analyte (unit)**	**n**	***r***	***P***
AST (U/L)	163	0.074	0.349
GGT (U/L)	153	0.139	0.087
CHOL (mmol/L)	70	0.071	0.559
TG (mmol/L)	83	0.563	<0.001*
HDL (mmol/L)	60	−0.153	0.244

Spearman's rank correlation coefficient was used to determine the AST, GGT, CHOL, TG, and HDL levels. *Statistically significant. Correlation analyses were performed using valid measurement data obtained after confirming the absence of lipemic interference, as described in the Methods section. The difference in numbers was due to the different datasets used in the correlation analysis compared with other analyses. Specifically, the correlation analysis in Table 4 required the LIP to be treated as a quantitative value. However, in routine laboratory practice, lipemia is typically qualitatively recorded. Quantitative values can only be obtained

by operating the measuring device directly on the day of measurement and quantifying the LIP. Consequently, only patients for whom quantitative values were obtained were included in the correlation analysis. Conversely, cases in which only qualitative values were obtained are analyzed separately in the other tables. This methodological difference led to discrepancies in the sample numbers.

AST, aspartate aminotransferase; GGT, gamma-glutamyl transferase; CHOL, total cholesterol; TG, triglycerides; HDL, high-density lipoprotein cholesterol.

Incidence of dyslipidemia and liver disease

To determine whether disease frequency was closely related to lipemia, the incidences of dyslipidemia and liver disease were examined in patients in the non-, low-, and high-lipemia groups. The data obtained showed that the proportion of individuals with dyslipidemia was relatively, but not statistically, higher in the low- and high-lipemia groups than in the non-lipemia group (non-lipemia group, 26.4%; low-lipemia group, 32.9%; high-lipemia group, 34.3%; low vs. non-lipemia groups, P = 0.189; high vs. non-lipemia groups, P = 0.129) (Fig. 1). The prevalence of liver disease did not differ significantly among the groups (non- vs. low-lipemia group, P = 0.979; and non-and high-lipemia groups, P = 0.905) (Fig. 1).

The proportion of individuals with both dyslipidemia and liver disease was significantly higher in the high-lipemia group than in the non-lipemia group (P = 0.024) (Fig. 1). In the non-

lipemia group, 4 of 108 (3.7%) individuals had dyslipidemia and liver disease. Of the four individuals, three had viral hepatitis B and one had steatotic liver disease (SLD) (Table 5). In the low-lipemia group, 16 of 246 (6.5%) patients had dyslipidemia and liver disease, including five patients with viral hepatitis B or C, five patients with SLD, two patients with liver diseases (unspecified), three patients with primary biliary cholangitis, and one patient with hepatic hemangioma (Table 5). In the high-lipemia group, 23 of 204 (11.3%) patients had dyslipidemia and liver disease, including six patients with hepatitis (viral hepatitis B, C, and unspecified), six patients with SLD, six patients with liver disease, two patients with cirrhosis (liver cirrhosis and primary biliary cholangitis), two patients with liver cancer (hepatocellular carcinoma and metastatic liver carcinoma), and one patient with liver cysts (Table 5).

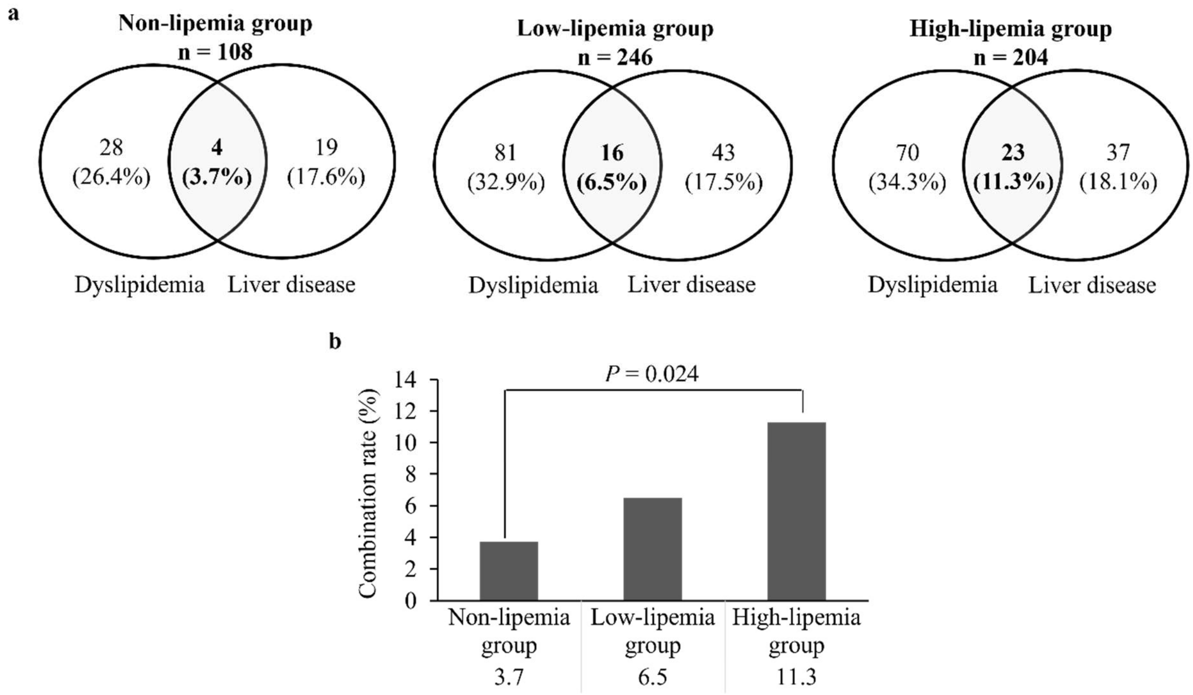

Figure 1. Rate of dyslipidemia and liver disease in each group

(a) Venn diagram illustrating the intersection between dyslipidemia and liver disease. Values are expressed as numbers (percentages). (b) Complication rates for dyslipidemia and liver disease

Table 5. Details of liver disease in individuals with dyslipidemia and liver disease

	**Non-lipemia (n=108)**	**Low-lipemia (n=246)**	**High-lipemia (n=204)**
Combination of dyslipidemia and liver disease	4	16	23
Hepatitis	**3**	**5**	**6**
Viral hepatitis B	3	3	2
Viral hepatitis C	0	2	2
Unspecified hepatitis	0	0	2
Steatotic liver disease	**1**	**5**	**6**
Liver disease, unspecified	**0**	**2**	**6**
Cirrhosis	**0**	**3**	**2**
Liver cirrhosis	0	0	1
Primary biliary cholangitis	0	3	1
Liver cancer	**0**	**0**	**2**
Hepatocellular carcinoma	0	0	1
Metastatic liver carcinoma	0	0	1
Hemangioma	**0**	**1**	**0**

Liver cyst	**0**	**0**	**1**

Data are expressed as n.

Liver disease subcategories are classified according to the ICD-10 (2019 version): Viral hepatitis B: acute hepatitis B (B16), chronic viral hepatitis B without delta-agent (B18.1); Viral hepatitis C: acute hepatitis C (B17.1), chronic viral hepatitis C (B18.2); Unspecified hepatitis: acute or chronic hepatitis, unspecified (B17.9, K73.9); Steatotic liver disease: fatty liver, not elsewhere classified (K76.0), alcoholic fatty liver (K70.0); Liver disease, unspecified (K76.9); Liver cirrhosis: other and unspecified cirrhosis of liver (K74.6); Primary biliary cholangitis: primary biliary cirrhosis (K74.3); hepatocellular carcinoma (C22.0); Metastatic liver carcinoma: secondary malignant neoplasm of liver and intrahepatic bile duct (C78.7); Hemangioma: hemangioma, any site (D18.0); Liver cyst: other specified diseases of liver (including simple cyst of liver) (K76.8).

Discussion

Lipemia is a common characteristic of both analytical plasma and serum samples. To investigate whether lipemic specimens were useful indicators of elevated clinical data or of non-lipidemic disorders, 27 sets of clinical chemical data and disease frequencies in individuals with lipemia were analyzed. Tian et al. reported that the prevalence of lipemic samples was higher in males than females [13]. In this study, the proportion of male patients was significantly

greater in the high-lipemia group than in the non-lipemia group (Table 1). Therefore, the etiologic characteristics of lipemia might be related to genetic and environmental factors, such as a fatty diet. The primary finding of this study was that lipemic samples measured significantly increased in terms of clinical laboratory data, in lipid metabolism-related parameters, and non-lipemic parameters (such as AST and GGT) (Tables 1, 3, and Supplementary Tables 2, 3). As expected, TG levels were significantly increased in lipemic specimens (Tables 1 and 2). CM and VLDL are the main constituents of TGs [1, 2]. Serum TG levels increase in individuals with lipemia [5, 7, 9, 10, 20–23]. According to Mainali et al., serum and plasma TG concentrations showed a weak correlation with LIP, as determined using an automated clinical chemistry analyzer ($r = 0.49$), with TG levels being significantly elevated in patients with lipemia [10]. Cobbold and Crook reported that the correlation between LIP and TG levels was 0.61 [22], suggesting that TG levels correlate with LIP. In support of these previous reports [10, 22, 23], the present study showed that TG levels increased significantly with increasing LIP levels. The median TG levels were 3.1- and 5.6-fold higher in the low- and high-lipemia groups, respectively (Table 1). The correlation between TG and LIP was 0.563 ($P < 0.001$) (Table 4), which was almost the same as previously reported [22]. In this study, HDL levels decreased in both the low- and high-lipemia groups were comparable to those in the non-lipemia group, whereas LDL values showed no significant differences among the groups (Table 1). CHOL values were significantly higher in the low- and high-lipemia groups than in the non-

lipemia group (Table 1 and 3). However, none of these parameters (HDL and CHOL) showed a statistically significant correlation with the LIP (HDL, $r = -0.153$, $P = 0.244$; CHOL, $r = 0.071$, $P = 0.559$) (Table 4). These findings support those of previous clinical studies that reported that elevated TG levels were closely associated with lower HDL levels [30], whereas LDL levels were not correlated with lipemia [7]. In contrast, Cobbold and Crook reported an inverse correlation between CHOL and LIP ($r = -0.41$) [22]. CHOL is the sum of lipoprotein cholesterol such as VLDL, LDL, and HDL. Therefore, it is plausible that the differences in results may be related to the different lipoprotein compositions of CHOL among individuals.

In this study, the possibility that lipemia could be correlated with elevated non-lipemic clinical parameters as well as lipid metabolism–related parameters was examined. Notably, liver chemistries, such as AST and GGT, were considerably higher in the high-lipemia group than in the non-lipemia group (Tables 1 and 3). These relationships were supported by the multinomial logistic regression analysis (Table 2). The levels of these parameters were not statistically proportional to the LIP values (Table 4); thus, qualitative, but not quantitative, LIP values may be useful for detecting individuals with liver chemistries above the reference interval.

To date, few studies have reported the frequency of non-lipidemic metabolic disorders in patients with lipemia. Several investigations have shown a higher rate of diabetes mellitus in individuals with lipemic specimens [1, 10], which differs from the present data. Although the

reason for these contradictory data remains unclear, the levels of Glu and HbA1c in the non-lipemia group were relatively higher than those of the reference interval (median Glu, 6.61 [IQR, 5.66–7.33] mmol/L; median HbA_{1c}, 44.27 [IQR, 37.71–49.73] mmol/mol) (Table 1). The prevalence of prediabetes and diabetes among the study participants was examined, and these groups were found to exhibit a higher than expected overall baseline prevalence of diabetes (non-, low-, and high-lipemia groups, 41.7%, 39.8%, and 41.7%, respectively). This elevated glycemic profile across the cohort may reflect the fact that the study population was recruited from a specialist hospital that plays a leading role in providing advanced medical care for patients with intractable diseases as well as for local residents. Consequently, the underlying metabolic burden in the normal plasma group may have obscured any differential impact of lipemic plasma on diabetes incidence, resulting in the observed non-significant difference (P = 0.909). The differing analytical data for Glu and HbA1c levels in non-lipidemic control participants among the studies may account for the variation in statistical outcomes. Further analyses are required to confirm the association between diabetes mellitus and lipemia.

Finally, the association between disease frequency and lipemia was examined as both liver chemistries (AST and GGT) and lipid profile parameters (CHOL and TG) increased in the lipemic samples in the present study. Although the prevalence of either dyslipidemia or liver disease alone did not differ significantly between the lipemia groups and the non-lipemia group, the coexistence of these two conditions showed a clear increase with higher degrees of

lipemia (Fig. 1). In the high-lipemia group, there was a significant increase in the rate of concurrent dyslipidemia and liver disease compared with that in the non-lipemia group (3.7% vs. 11.3%; $P = 0.024$). The reason for the high co-occurrence of both diseases among individuals with lipemia remains unknown. Postprandial lipemia occurs when the levels of both intestinal-derived CM and liver-derived VLDL increase [33]. In patients with liver disease, serum hypertriglyceridemia is partly caused by a decrease in plasma hepatic triglyceride lipase activity [34]. Lipemia associated with liver disease may result from more complex factors than postprandial conditions, and further analyses of the underlying mechanisms are necessary.

Dyslipidemia, such as hypertriglyceridemia, is associated with an increased risk of hepatic steatosis and fatty liver disease [35]. In this study, dyslipidemia was defined according to the JAS clinical practice guidelines [29]. The lipid thresholds defined in the JAS guidelines differ from those described in the National Cholesterol Education Program Adult Treatment Panel (ATP) III report [36]. The JAS provides diagnostic thresholds (e.g., LDL ≥3.62 mmol/L and TG ≥1.69 mmol/L), whereas ATP III uses several lipid parameters for risk stratification and treatment decision-making rather than for diagnostic purposes. The JAS guidelines were considered appropriate for the present study population because they reflect the epidemiological evidence and cardiovascular risk characteristics specific to the Japanese population. Although the differences between the JAS and ATP III criteria may limit direct

comparisons with studies conducted in other countries, the JAS thresholds offer a suitable framework for interpreting lipid abnormalities in Japanese cohorts. Recently, the term metabolic dysfunction-associated SLD (MASLD) was introduced for patients with chronic SLD who exhibit at least one cardiometric factor and no other identifiable causes of steatosis [35]. The cardiometabolic criteria for MASLD include the presence of dyslipidemia (TG ≥1.70 mmol/L or lipid-lowering treatment, HDL ≤1.0 mmol/L for males and HDL ≤1.3 mmol/L for females or lipid-lowering treatment) [35]. MASLD has become increasingly prevalent worldwide. A large cohort study by Perazzo et al. revealed that MASLD was observed in 3,569 of 10,651 Brazilian patients, accounting for 33.5% of cases [37]. Song et al. examined 1,016 individuals who underwent proton magnetic resonance spectroscopy in Hong Kong and reported that 271 (26.7%) had MASLD [38]. Considering the higher morbidity rate of SLD [37, 38], many clinicians have proposed that assessing the severity of SLD is important for preventing and treating SLD-related complications. The FLI serves as a diagnostic algorithm for SLD, incorporating body mass index, waist circumference, and serum concentrations of GGT and TG [15]. Several studies have reported that FLI may be useful for predicting other diseases, such as diabetes mellitus, chronic kidney disease, and ischemic heart disease [39–41]. Nevertheless, other studies have reported that FLI has limited utility in diagnosing liver steatosis [42, 43], and the diagnostic value of FLI in SLD remains controversial. Based on this evidence, it would be clinically valuable to develop a tool that is

more appropriate for identifying patients at risk for dyslipidemia associated with liver dysfunction. Unlike the FLI, which requires the manual calculation of four variables, the LIP is automatically generated during routine laboratory processing, potentially enabling real-time MASLD risk assessment. It might be intriguing to explore whether the LIP (a routinely available laboratory parameter to detect turbidity due to lipemia) might serve as a surrogate indicator that captures broader aspects of lipid-related metabolic disturbance, including hepatic steatosis. The clinical management of atherosclerotic disease risk depends crucially on measuring blood lipid and lipoprotein levels [44, 45], and lipemia is commonly considered a potential contributor enhancing the risk of cardiovascular disease (CVD) [46]. This study was conducted at a university-affiliated tertiary care hospital. Consequently, the patient population may differ from that seen in general health checkup centers. More extensive studies are recommended to investigate whether lipemia is useful in assessing the risk of liver diseases such as MASLD.

Study Strengths and Limitations

This study had several notable strengths. First, this was the first study to comprehensively evaluate a wide range of clinical chemistry and hematological parameters in both non-lipemic and lipemic groups to examine their association with disease. This analysis included 27 commonly available clinical laboratory analytes, enabling a broad assessment of systemic

metabolic alterations beyond lipid metabolism. To date, few studies have investigated the relationship between lipemia and the risk of diverse diseases, including those not directly related to dyslipidemia. Thus, the clinical significance of lipemic plasma characteristics is reinforced by the present results. Second, the use of multinomial logistic regression adjusted for age and sex enhanced the robustness of the results by reducing potential confounding factors. Third, lipemia was objectively assessed using an automated clinical chemistry analyzer to ensure reproducibility and to minimize the observer bias. Unlike studies that have relied solely on the visual inspection of plasma turbidity, this study provided a quantitative LIP parameter, which enabled robust comparisons across patient groups. Fourth, although previous studies have primarily focused on lipid abnormalities, this study uniquely examined the association of lipemia with liver chemistries, concomitant dyslipidemia, and liver disease. Given the well-recognized mechanistic links between dyslipidemia and lipid metabolism–related liver diseases, such as MAFLD, the findings not only reinforce this correlation but also highlight the potential clinical utility of LIP as a novel biomarker of lipid and hepatic pathophysiology.

This study also had some limitations. First, all participants, including those in the non-lipidemic group, were recruited from the same advanced treatment hospital. Due to the limited availability of clinical control data from participants without lipemia, the comparison of laboratory data between the non-lipemic and lipemic groups may be biased. Because the non-lipemia control group was randomly selected from individuals without lipemia, the age and sex

distribution differed from that of the lipemia group. Although age and sex were adjusted for in a multinomial model, these variables are established risk factors for dyslipidemia and liver dysfunction, and therefore residual confounding cannot be entirely ruled out. Thus, large-scale, multi-institutional, randomized controlled trials are required to validate the present findings and evaluate the effectiveness of measuring the degree of lipemia. Second, the validity of the lipemia classification system used in this study was self-reported and not verified by others. Among the different methods for measuring lipemia levels in samples, LIP measurement using an automated chemical analyzer is a convenient and reproducible method for analyzing lipemia. Currently, no standardized method exists for determining the LIP among the manufacturers of automated chemical analyzers. Lipemia is identifiable across a wide wavelength range, from 300 to 700 nm, and different manufacturers use different wavelengths to detect LIP [2]. This study utilized the LIP derived from the analytical results of measuring emissions across multiple wavelengths, and it was calculated based on INTRAFAT® injection of intravenous fat (soybean oil; Takeda Pharmaceutical Co., Ltd., Osaka, Japan) as a reference material, in a manner consistent with the approaches described in the Clinical Laboratory Standards Institute (CLSI) guidelines C56-A [47]. Participants were categorized as having non- (normal; <1.5 LIP), low- (+1–2; 1.5–4.9 LIP), and high- (+3–4; ≥5.0 LIP) lipemia levels. Future research should either develop standardized protocols based on findings such as those reported here or propose a harmonized procedure to improve comparability across different analytical platforms. Third,

this study collected laboratory data and diagnosed patients with dyslipidemia; however, detailed information regarding their treatment was not available. Information on medications known to cause severe hypertriglyceridemia and chylomicronemia (including L-asparaginase, mTOR inhibitors, and antiretroviral protease inhibitors) was unavailable. Although this may represent a source of residual confounding, the likelihood of substantial bias is considered low given the rarity of such therapies in this study population. It is difficult to completely rule out the possibility that cases involving non-fasting individuals might affect the retrospective analyses [10, 48]. Future prospective studies should systematically collect information on fasting status and medications to better account for the factors influencing lipemia. However, it is evident from the results of this study that a significant increase in the prevalence of concomitant dyslipidemia and liver disease was observed among participants with high lipid levels. Therefore, measuring the extent of lipemia can be helpful even when data are only partially available.

Conclusions

Lipemia results from elevated levels of CM and VLDL and is recognized as a potential risk factor for CVD. The present study indicates that lipemia is linked to increased levels of lipid metabolism markers (CHOL and TG) and irregularities in non-lipid metabolic clinical chemistry parameters, including liver chemistries. AST and GGT levels were significantly

elevated in the high-lipemia group (≥5.0 LIP) compared with those of the non-lipemia group. Moreover, the high-lipemia group exhibited a greater prevalence of concurrent liver disease and dyslipidemia. Individuals with lipemic specimens can be easily identified using an automated clinical analyzer. Therefore, assessing lipemia levels may serve as a practical approach for identifying individuals with non-lipid-related conditions, including liver disease. This study suggests that lipemia is associated not only with lipid abnormalities but also with liver chemistries, dyslipidemia, and liver disease. The routine recognition of lipemia during automated biochemical testing could alert clinicians to possible underlying liver dysfunction or metabolic comorbidities. Future research should clarify whether leveraging the LIP values automatically generated as ancillary information in routine laboratory testing could help identify high-risk patients earlier and contribute to stratify their risk of metabolic-hepatic comorbidities.

Abbreviations

CM	Chylomicrons
VLDL	Very low-density lipoprotein
TG	Triglyceride
HIV	Human immunodeficiency virus
LPL	Lipoprotein lipase

MASLD	Metabolic dysfunction-associated SLD
FLI	Fatty liver index
LIP	Lipemia index
FTU	Formazin turbidity unit
JAS	Japan Atherosclerosis Society
TP	Total protein
Alb	Albumin
CREA	Creatinine
UA	Uric acid
Na	Sodium
K	Potassium
Cl	Chlorine
Ca	Calcium
AST	Aspartate aminotransferase
ALT	Alanine aminotransferase
AMY	Amylase
TBIL	Total bilirubin
CHE	Cholinesterase
GGT	Gamma-glutamyltransferase

CHOL	Total cholesterol
HDL	High-density lipoprotein cholesterol
LDL	Low-density lipoprotein cholesterol
ALP	Alkaline phosphatase
Glu	Glucose
HbA_{1c}	Glycated hemoglobin
WBC	White blood cells
RBC	Red blood cells
Hb	Hemoglobin
Hct	Hematocrit
Plt	Platelets
IQR	Interquartile range
SLD	Steatotic liver disease
ICD-10	International Classification of Diseases, 10th Revision
ATP	Adult Treatment Panel
CVD	Cardiovascular disease
CLSI	Clinical laboratory standards institute

Declarations

Ethics approval and consent to participate

This study was approved by the Ethics Committee of Niigata University on 30 June 2022 (Approval number: 2022-0035). As this study used clinical analytes obtained during routine medical care and did not involve the collection or testing of new specimens, the requirement for informed consent was waived. The participants were provided with the option to opt out of the study if they declined to participate.

Consent for publication

Not applicable.

Availability of data and materials

The data underlying the findings of this study are available from the Niigata University Medical and Dental Hospital. However, access is restricted by licensing agreements, and the data are not publicly available. Interested researchers may request access from the corresponding author, subject to reasonable justification and approval from Niigata University Medical and Dental Hospital.

Competing interests

The authors declare no competing interests.

Funding

This study was funded by a Grant-in-Aid for the Encouragement of Scientists from the Japan Society for the Promotion of Science [grant number 22H04387]. The funding organization was not involved in the study design, data collection, analysis, decision to publish, or manuscript preparation.

Author contributions

M.O.: conceptualization, study design, data analysis, data curation, funding acquisition, and writing (original draft, review, and editing).

Y.M.: Data analysis and writing—review and editing.

T.U.: Data analysis and writing—review and editing.

T.W.: Data collection and writing—review and editing.

All authors have reviewed and approved the final manuscript for publication.

Acknowledgments

We sincerely appreciate the English language editing support provided by Editage, a division of CACTUS Communications.

References

1. Garvey WT, Kwon S, Zheng D, Shaughnessy S, Wallace P, Hutto A, et al. Effects of insulin resistance and type 2 diabetes on lipoprotein subclass particle size and concentration determined by nuclear magnetic resonance. Diabetes. 2003;52:453-62.

2. Nikolac N. Lipemia: causes, interference mechanisms, detection and management. Biochem Med. 2014;24:57-67.

3. Park Y, Grellner WJ, Harris WS, Miles JM. A new method for the study of chylomicron kinetics in vivo. Am J Physiol Endocrinol Metab. 2000;279:E1258-63.

4. Kroll MH. Evaluating interference caused by lipemia. Clin Chem. 2004;50:1968-9.

5. Cohen JC, Berger GM. Effects of glucose ingestion on postprandial lipemia and triglyceride clearance in humans. J Lipid Res. 1990;31:597-602.

6. van Tol A, van der Gaag MS, Scheek LM, van Gent T, Hendriks HF. Changes in postprandial lipoproteins of low and high density caused by moderate alcohol consumption with dinner. Atherosclerosis. 1998;141(suppl. 1):S101-3.

7. Tinker LF, Parks EJ, Behr SR, Schneeman BO, Davis PA. (n-3) fatty acid supplementation in moderately hypertriglyceridemic adults changes postprandial lipid and apolipoprotein B responses to a standardized test meal. J Nutr. 1999;129:1126-34.

8. Jackson KG, Robertson MD, Fielding BA, Frayn KN, Williams CM. Olive oil increases the number of triacylglycerol-rich chylomicron particles compared with other oils: an effect retained when a second standard meal is fed. Am J Clin Nutr. 2002;76:942-49.

9. Lim K-H, Lian W-B, Yeo C-L. Does visual turbidity correlate with serum triglyceride levels in babies on total parenteral nutrition? Ann Acad Med Singap. 2006;35:790-3.

10. Mainali S, Davis SR, Krasowski MD. Frequency and causes of lipemia interference of clinical chemistry laboratory tests. Pract Lab Med. 2017;8:1-9.

11. Sen Gupta P, Sharma M, Timms PM. Laboratory samples deemed 'unsuitable for analysis' can be diagnostically useful. Clin Med (Lond). 2013;13:309-11.

12. Burnett JR, Hooper AJ, Hegele RA. Familial lipoprotein lipase deficiency. In: Adam MP, Ardinger HH, Pagon RA, Wallace SE, Bean LJ, Stephens K, Amemiya A, eds. GeneReviews® [Internet]. University of Washington: Seattle, WA, 1993 https://www.ncbi.nlm.nih.gov/books/NBK1308/.

13. Tian G, Wu Y, Jin X, Zeng Z, Gu X, Li T, et al. The incidence rate and influence factors of hemolysis, lipemia, icterus in fasting serum biochemistry specimens. PLOS One. 2022;17:e0262748.

14. Ng PC, Lam CW, Fok TF, Lee CH, Lo DY, Chan LY, et al. Deceptive hyperbilirubinaemia in a newborn with familial lipoprotein lipase deficiency. J Paediatr Child Health. 2001;37:314-6.

15. Bedogni G, Bellentani S, Miglioli L, Masutti F, Passalacqua M, Castiglione A, Tiribelli C. The fatty liver index: a simple and accurate predictor of hepatic steatosis in the general population. BMC Gastroenterol. 2006;6:33.

16. Rinella ME, Neuschwander-Tetri BA, Siddiqui MS, Abdelmalek MF, Caldwell S, Barb D et al. AASLD Practice Guidance on the clinical assessment and management of nonalcoholic fatty liver disease. Hepatology. 2023;77:1797-835.

17. Havel RJ, Gordon RS Jr. Idiopathic hyperlipemia: metabolic studies in an affected family. J Clin Invest. 1960;39:1777-90.

18. Knudsen P, Eriksson J, Lahdenperä S, Kahri J, Groop L, Taskinen MR. Changes of lipolytic enzymes cluster with insulin resistance syndrome. Botnia Study Group. Diabetologia. 1995;38:344-50.

19. Nishizawa Y, Shoji T, Nishitani H, Yamakawa M, Konishi T, Kawasaki K, et al. Hypertriglyceridemia and lowered apolipoprotein c-II/c-III ratio in uremia: effect of a fibric acid, clinofibrate. Kidney Int. 1993;44:1352-1359.

20. De Haene H, Taes Y, Christophe A, Delanghe J. Comparison of triglyceride concentration with lipemic index in disorders of triglyceride and glycerol metabolism. Clin Chem Lab Med. 2006;44:220-2.

21. Ooi TC, Robinson L, Graham T, Kolovou GD, Mikhailidis DP, Lairon D. Proposing a 'lipemic index' as a nutritional and research tool. Curr Vasc Pharmacol. 2011;9:313-7.

22. Cobbold L, Crook MA. The lipaemic index: clinical observations. Br J Biomed Sci. 2015;72:52-5.

23. Van Elslande J, Hijjit S, De Vusser K, Langlois M, Meijers B, Mertens A, et al. Delayed diagnosis and treatment of extreme hypertriglyceridemia due to rejection of a lipemic sample. Biochem Med. 2021;31:021002.

iThenticate Page 43 of 71 - Integrity Submission Submission ID trn:oid:::3117:535726404

24. Agrawal YP, Hall K. The Lipemia Index: an underutilized tool to detect monoclonal proteins. J Appl Lab Med. 2019;3:1062-4.

25. Shin DH, Kim J, Uh Y, Lee SI, Seo DM, Kim KS, et al. Development of an integrated reporting system for verifying hemolysis, icterus, and lipemia in clinical chemistry results. Ann Lab Med. 2014;34:307-12.

26. Cadamuro J, Lippi G, von Meyer A, Ibarz M, van Dongen-Lases E, Cornes M, et al. European survey on preanalytical sample handling—Part 2: Practices of European laboratories on monitoring and processing haemolytic, icteric and lipemic samples. Biochem Med (Zagreb). 2019;29:334-45.

27. Fernández Prendes C, Castro Castro MJ, Sánchez Navarro L, Rapún Mas L, Morales Indiano C, Arrobas Velilla T. Handling of lipemic samples in the clinical laboratory. Adv Lab Med. 2023;4:5-27.

28. Kristoffersen AH, Hollestelle MJ, Cadamuro J, Hillarp A, Jennings I, Marrington R, et al. Practical handling of hemolytic, icteric and lipemic samples for coagulation testing in European laboratories. A collaborative survey from the European Organisation for External Quality Assurance Providers in Laboratory Medicine (EQALM). Clin Chem Lab Med. 2025;63:2074-84.

29. Okamura T, Tsukamoto K, Arai H, Fujioka Y, Ishigaki Y, Koba S, et al. Japan Atherosclerosis Society (JAS) Guidelines for Prevention of Atherosclerotic Cardiovascular Diseases 2022. J Atheroscler Thromb. 2023;31:641-853.

30. Kwo PY, Cohen SM, Lim JK. ACG Clinical Guideline: evaluation of abnormal liver chemistries. Am J Gastroenterol. 2017;112:18-35.

31. Japanese Committee for Clinical Laboratory Standards (JCCLS). Japanese shared reference intervals: reference intervals for major clinical laboratory tests in Japan. 2022 ed. Tokyo: JCCLS; 2022 [in Japanese].

32. Syvänne M, Taskinen MR. Lipids and lipoproteins as coronary risk factors in non-insulin-dependent diabetes mellitus. Lancet. 1997;350(suppl. 1):SI20-3.

33. Nakajima K, Nakano T, Tokita Y, Nagamine T, Inazu A, Kobayashi J, et al. Postprandial lipoprotein metabolism: VLDL vs chylomicrons. Clin Chim Acta. 2011;412:1306-18.

34. Klose G, Windelband J, Weizel A, Greten H. Secondary hypertriglyceridaemia in patients with parenchymal liver disease. Eur J Clin Investig. 1977;7:557-62.

35. Rinella ME, Lazarus JV, Ratziu V, Francque SM, Sanyal AJ, Kanwal F, et al. A multisociety Delphi consensus statement on new fatty liver disease nomenclature. J Hepatol. 2023;79:1542-56.

36. National Cholesterol Education Program (NCEP) Expert Panel on Detection, Evaluation, and Treatment of High Blood Cholesterol in Adults (Adult Treatment Panel III). Third Report of the National Cholesterol Education Program (NCEP) Expert Panel on Detection, Evaluation, and Treatment of High Blood Cholesterol in Adults (Adult Treatment Panel III) Final Report. Circulation. 2002;106:3143-421.

37. Perazzo H, Pacheco AG, Griep RH, Collaborators. Changing from NAFLD through MAFLD to MASLD: similar prevalence and risk factors in a large Brazilian cohort. J Hepatol. 2024;80:e72-4.

38. Song SJ, Lai JC, Wong GL, Wong VW, Yip TC. Can we use old NAFLD data under the new MASLD definition? J Hepatol. 2024;80:e54-6.

39. Nishi T, Babazono A, Maeda T, Imatoh T, Une H. Evaluation of the fatty liver index as a predictor for the development of diabetes among insurance beneficiaries with prediabetes. J Diabetes Investig. 2015;6:309-16.

40. Huh JH, Kim JY, Choi E, Kim JS, Chang Y, Sung K-C. The fatty liver index as a predictor of incident chronic kidney disease in a 10-year prospective cohort study. PLOS One. 2017;12:e0180951.

41. Niu Y, Wang G, Feng X, Niu H, Shi W. Significance of fatty liver index to detect prevalent ischemic heart disease: evidence from national health and nutrition examination survey 1999-2016. Front Cardiovasc Med. 2023;10:1171754.

42. Borman MA, Ladak F, Crotty P, Pollett A, Kirsch R, Pomier-Layrargues G, et al. The Fatty Liver Index has limited utility for the detection and quantification of hepatic steatosis in obese patients. Hepatol Int. 2013;7:592-9.

43. Lajeunesse-Trempe F, Boit MK, Kaduka LU, De Lucia-Rolfe E, Baass A, Paquette M, et al. Validation of the Fatty Liver Index for identifying non-alcoholic fatty liver disease in a Kenyan population. Trop Med Int Health. 2023;28:830-8.

44. Laufs U, Parhofer KG, Ginsberg HN, Hegele RA. Clinical review on triglycerides. Eur Heart J. 2020;41:99-109c.

45. Cao J, Donato L, El-Khoury JM, Goldberg A, Meeusen JW, Remaley AT. ADLM guidance document on the measurement and reporting of lipids and lipoproteins. J Appl Lab Med. 2024;9:1040-56.

46. Jackson KG, Poppitt SD, Minihane AM. Postprandial lipemia and cardiovascular disease risk: interrelationships between dietary, physiological and genetic determinants. Atherosclerosis. 2012;220:22-33.

47. Clinical Laboratory Standards Institute, 2012. Hemolysis, icterus, and lipemia/turbidity indices as indicators of interference in clinical laboratory analysis; Approved Guideline CLSI C56-A document. Clinical Laboratory Standards Institute: Wayne, PA, USA.

48. Cartier L-J, Collins C, Lagacé M, Douville P. Comparison of fasting and non-fasting lipid profiles in a large cohort of patients presenting at a community hospital. Clin Biochem. 2018;52:61-6.

Table 1. Data and statistical analysis for each analyte in the non-, low-, and high-lipemia groups

Analyte (unit)	n	Non-lipemia group (n = 108)	Low-lipemia group (n = 246)	High-lipemia group (n = 204)	*P*	*P*		
						Non- vs. low-lipemia group	Non- vs. high-lipemia group	Low- vs. high-lipemia group
Age (years)	558	68 [55–75]	59 [49–68]	54 [46–65]	<0.001	0.001*	<0.001*	0.060
Sex, male	558	54 (50.0)	157 (63.8)	147 (72.1)	<0.001 †	0.015*	<0.001*	0.063
TP (g/L)	466	72 [69–75]	71 [67–75]	71 [68–75]	0.277	NA	NA	NA
(g/dL)		7.2 [6.9–7.5]	7.1 [6.7–7.5]	7.1 [6.8–7.5]				

Alb (g/L)	444	41 [39–43]	40 [38–43]	41 [39–43]	0.236	NA	NA	NA
(g/dL)		4.1 [3.9–4.3]	4.0 [3.8–4.3]	4.1 [3.9–4.3]				
Urea (mmol/L)	527	5.71 [4.64–6.43]	5.71 [4.64–7.14]	5.71 [4.64–7.14]	0.357	NA	NA	NA
(mg/dL)		16 [13–18]	16 [13–20]	16 [13–20]				
CREA (µmol/L)	544	68.07 [59.23–84.86]	76.02 [61.00–91.05]	77.79 [63.21–95.03]	0.027	0.277	0.022*	0.563
(mg/dL)		0.77 [0.67–0.96]	0.86 [0.69–1.03]	0.88 [0.72–1.08]				
UA (µmol/L)	385	0.31±0.09	0.33±0.09	0.36±0.10	<0.001 ‡	0.227	<0.001*	0.006*

(mg/dL)		5.2±1.5	5.5±1.5	6.1±1.7				
Na (mmol/L)	509	140 [139–142]	141 [139–142]	140 [138–141]	<0.001	0.585	0.078	0.000*
K (mmol/L)	507	4.2 [3.8–4.4]	4.1 [3.8–4.4]	4.0 [3.8–4.3]	0.045	0.488	0.042*	0.473
Cl (mmol/L)	498	105 [103–106]	104 [102–106]	104 [102–106]	0.049	0.250	0.043*	0.959
Ca (mmol/L)	352	2.32 [2.27–2.40]	2.32 [2.27–2.40]	2.35 [2.30–2.42]	0.023	1.000	0.070	0.058
(mg/dL)		9.3 [9.1–9.6]	9.3 [9.1–9.6]	9.4 [9.2–9.7]				

AST (U/L)	520	23 [20–28]	25 [21–32]	27 [22–35]	0.003	0.108	0.002*	0.237
ALT (U/L)	520	19 [15–27]	23 [16–33]	25 [17–41]	0.006	0.053	0.004*	0.766
AMY (U/L)	263	79 [61–101]	88 [62–119]	73 [61–95]	0.034	0.410	1.000	0.034*
TBIL (μmol/L)	478	11.97 [8.55–13.68]	10.26 [6.84–13.68]	8.55 [6.84–11.97]	<0.001	<0.001*	<0.001*	0.441
(mg/dL)		0.7 [0.5–0.8]	0.6 [0.4–0.8]	0.5 [0.4–0.7]				
CHE (U/L)	309	307±83	321±96	368±99	<0.001 ‡	0.618	<0.001*	<0.001*

GGT (U/L)	500	24 [16–40]	33 [20–66]	39 [26–79]	<0.001	0.004*	<0.001*	0.035*
CHOL (mmol/L)	230	5.15 [4.45–5.72]	5.66 [4.86–6.57]	5.83 [5.13–7.00]	<0.001	0.004*	<0.001*	1.000
(mg/dL)		199 [172–221]	219 [188–254]	226 [198–271]				
TG (mmol/L)	281	1.37 [0.91–1.83]	4.18 [2.81–5.76]	7.62 [5.72–11.29]	<0.001	<0.001*	<0.001*	<0.001*
(mg/dL)		122 [81–162]	370 [249–510]	674 [507–999]				
HDL (mmol/L)	213	1.45 [1.19–1.68]	1.23 [0.98–1.47]	0.91 [0.75–1.16]	<0.001	0.029*	<0.001*	<0.001*
(mg/dL)		56 [46–65]	48 [38–57]	35 [29–45]				

LDL (mmol/L)	218	2.89±0.77	3.13±0.98	2.69±1.00	0.014‡	0.307	0.442	0.010*
(mg/dL)		112±30	121±38	104±39				
ALP (U/L)	499	73 [56–87]	79 [61–98]	81 [65–96]	0.013	0.049*	0.012*	1.000
Glu (mmol/L)	82	6.61 [5.66–7.33]	6.33 [5.75–8.72]	6.52 [5.50–7.58]	0.711	NA	NA	NA
(mg/dL)		119 [102–132]	114 [104–157]	118 [99–137]				
HbA_{1c} (mmol/mol)	194	44.27 [37.71–49.73]	45.36 [38.53–54.38]	46.45 [38.80–58.48]	0.456	NA	NA	NA
(%)		6.2 [5.6–6.7]	6.3 [5.7–7.1]	6.4 [5.7–7.5]				

WBC (×10^9/L)	513	4.93 [4.05–6.10]	6.41 [4.92–7.92]	6.75 [5.27–8.50]	<0.001	<0.001*	<0.001*	0.328
(×10^3/μL)		4.93 [4.05–6.10]	6.41 [4.92–7.92]	6.75 [5.27–8.50]				
RBC (×10^12/L)	511	4.38 [4.06–4.77]	4.37 [3.79–4.75]	4.51 [4.02–4.89]	0.050	NA	NA	NA
(×10^6/μL)		4.38 [4.06–4.77]	4.37 [3.79–4.75]	4.51 [4.02–4.89]				
Hb (g/L)	511	132 [122–143]	134 [116–147]	143 [127–152]	<0.001	1.000	0.005*	0.001*
(g/dL)		13.2 [12.2–14.3]	13.4 [11.6–14.7]	14.3 [12.7–15.2]				
Hct (L/L)	512	0.40 [0.37–0.43]	0.41 [0.36–0.43]	0.42 [0.38–0.45]	0.009	1.000	0.119	0.009*

(%)		40.4 [37.2–43.0]	40.8 [35.5–43.4]	42.1 [38.3–45.1]				
Plt ($\times 10^9$/L)	511	201 [168–241]	226 [179–274]	233 [183–285]	0.001	0.009*	0.001*	1.000
($\times 10^3$/μL)		201 [168–241]	226 [179–274]	233 [183–285]				

Data are expressed as median [IQR: 25%–75%], n (%), or mean and standard deviation.

Both the SI and conventional units are presented. Statistical analyses were performed using the Kruskal–Wallis test with post hoc Dunn-Bonferroni test, †chi-square test, ‡one-way analysis of variance with post hoc Tukey's test. *Statistically significant. The total number of participants (N) varied for different analytes (Table 1) because not all participants underwent all 27 laboratory tests; analyses were performed using the available data for each parameter.

TP, total protein; NA, not analyzed; Alb, albumin; CREA, creatinine; UA, uric acid; Na, sodium; K, potassium; Cl, chloride; Ca, calcium; AST, aspartate aminotransferase; ALT, alanine aminotransferase; AMY, amylase; TBIL, total bilirubin; CHE, cholinesterase; GGT, gamma-glutamyltransferase; CHOL, total cholesterol; TG, triglycerides; HDL, high-density lipoprotein cholesterol; LDL, low-density lipoprotein

cholesterol; ALP, alkaline phosphatase; Glu, glucose; HbA_{1c}, glycated hemoglobin; WBC, white blood cells; RBC, red blood cells; Hb, hemoglobin; Hct, hematocrit; Plt, platelets

Table 2. Relationship between lipemia and each variable with multinomial logistic regression

Variables	Multinomial logistic regression (combining each variable with age and sex)			
	Low-lipemia group (n = 246)		High-lipemia group (n = 204)	
	OR (95% CI)	*P*	OR (95% CI)	*P*
TP (g/L, OR per 10 units)	0.62 (0.40–0.97)	0.035*	0.65 (0.41–1.03)	0.068
TP (g/dL, OR per 10 units)	0.62 (0.40–0.97)	0.035*	0.65 (0.41–1.03)	0.068
Alb (g/L, OR per 10 units)	0.71 (0.40–1.27)	0.248	0.72 (0.39–1.32)	0.283
Alb (g/dL, OR per 10 units)	0.71 (0.40–1.27)	0.248	0.72 (0.39–1.32)	0.283
Urea (mmol/L, OR per 1 units)	1.21 (1.07–1.36)	0.003*	1.21 (1.07–1.37)	0.002*
Urea (mg/dL, OR per 5 units)	1.40 (1.12–1.74)	0.003*	1.41 (1.13–1.76)	0.002*
CREA (μmol/L, OR per 10 units)	1.03 (0.99–1.08)	0.152	1.04 (0.99–1.08)	0.133
CREA (mg/dL, OR per 0.1 units)	1.03 (0.99–1.07)	0.152	1.03 (0.99–1.07)	0.133
UA (μmol/L, OR per 0.1 units)	1.19 (0.85–1.65)	0.311	1.75 (1.23–2.49)	0.002*
UA (mg/dL, OR per 1 units)	1.11 (0.91–1.35)	0.311	1.39 (1.13–1.72)	0.002*
Na (mmol/L, OR per 1 units)	1.06 (0.96–1.16)	0.249	0.93 (0.84–1.02)	0.105
K (mmol/L, OR per 1 units)	0.82 (0.46–1.45)	0.485	0.63 (0.34–1.17)	0.142
Cl (mmol/L, OR per 1 units)	0.95 (0.89–1.02)	0.140	0.93 (0.86–1.00)	0.044*

Ca (mmol/L, OR per 0.1 units)	1.02 (0.80–1.30)	0.849	1.38 (1.05–1.83)	0.021*
Ca (mg/dL, OR per 1 units)	1.06 (0.58–1.94)	0.849	2.25 (1.13–4.50)	0.021*
AST (U/L, OR per 10 units)	1.28 (1.01–1.63)	0.039*	1.45 (1.09–1.92)	0.010*
ALT (U/L, OR per 10 units)	1.15 (1.00–1.32)	0.054	1.09 (0.95–1.26)	0.220
AMY (U/L, OR per 10 units)	1.14 (1.02–1.27)	0.019*	1.05 (0.94–1.18)	0.389
TBIL (μmol/L, OR per 1 units)	1.00 (0.99–1.01)	0.477	0.99 (0.96–1.02)	0.478
TBIL (mg/dL, OR per 0.1 units)	1.01 (0.99–1.02)	0.477	0.98 (0.93–1.03)	0.478
CHE (U/L, OR per 100 units)	1.21 (0.85–1.73)	0.287	1.99 (1.36–2.90)	<0.001*
GGT (U/L, OR per 10 units)	1.06 (1.00–1.13)	0.062	1.09 (1.02–1.16)	0.012*
CHOL (mmol/L, OR per 1 units)	1.71 (1.25–2.33)	<0.001*	2.10 (1.51–2.92)	<0.001*
CHOL (mg/dL, OR per 10 units)	1.15 (1.06–1.25)	<0.001*	1.21 (1.11–1.32)	<0.001*
TG (mmol/L, OR per 1 units)	3.60 (2.43–5.32)	<0.001*	5.23 (3.47–7.87)	<0.001*
TG (mg/dL, OR per 10 units)	1.16 (1.11–1.21)	<0.001*	1.21 (1.15–1.26)	<0.001*
HDL (mmol/L, OR per 1 units)	0.54 (0.25–1.18)	0.124	0.11 (0.04–0.28)	<0.001*
HDL (mg/dL, OR per 10 units)	0.85 (0.70–1.04)	0.124	0.56 (0.43–0.72)	<0.001*
LDL (mmol/L, OR per 1 units)	1.36 (0.93–1.99)	0.116	0.82 (0.55–1.23)	0.343
LDL (mg/dL, OR per 10 units)	1.08 (0.98–1.19)	0.116	0.95 (0.86–1.06)	0.343

ALP (U/L, OR per 10 units)	1.09 (1.00–1.19)	0.040*	1.09 (1.00–1.19)	0.042*
Glu (mmol/L, OR per 1 units)	1.24 (0.85–1.81)	0.262	1.09 (0.74–1.59)	0.662
Glu (mg/dL, OR per 10 units)	1.13 (0.92–1.39)	0.262	1.05 (0.85–1.30)	0.662
HbA_{1c} (mmol/mol, OR per 10 units)	1.34 (0.91–1.98)	0.134	1.44 (0.97–2.14)	0.070
HbA_{1c} (%, OR per 1 units)	1.38 (0.91–2.11)	0.134	1.49 (0.97–2.29)	0.070
WBC ($\times 10^9$/L, OR per 1 units)	1.36 (1.20–1.53)	<0.001*	1.33 (1.18–1.50)	<0.001*
WBC ($\times 10^3$/L, OR per 1000 units)	1.03 (1.02–1.05)	<0.001*	1.03 (1.02–1.04)	<0.001*
RBC ($\times 10^{12}$/L, OR per 1 units)	0.66 (0.46–0.95)	0.025*	0.84 (0.57–1.23)	0.362
RBC ($\times 10^6$/L, OR per 100 units)	0.66 (0.46–0.95)	0.025*	0.84 (0.57–1.23)	0.362
Hb (g/L, OR per 10 units)	0.92 (0.82–1.03)	0.149	1.01 (0.90–1.14)	0.839
Hb (g/dL, OR per 1 units)	0.92 (0.82–1.03)	0.149	1.01 (0.90–1.14)	0.839
Hct (L/L, OR per 0.1 units)	0.65 (0.43–0.98)	0.041*	0.96 (0.64–1.46)	0.854
Hct (%, OR per 10 units)	0.65 (0.43–0.98)	0.041*	0.96 (0.64–1.46)	0.854
Plt ($\times 10^9$/L, OR per 100 units)	1.40 (1.01–1.94)	0.045*	1.59 (1.12–2.24)	0.009*
Plt ($\times 10^3$/L, OR per 10 units)	1.40 (1.01–1.94)	0.045*	1.59 (1.12–2.24)	0.009*

*Statistically significant. OR, odds ratio; CI, confidence interval; TP, total protein; Alb, albumin; CREA, creatinine; UA, uric acid; Na, sodium; K, potassium; Cl, chloride; Ca, calcium; AST, aspartate aminotransferase; ALT, alanine aminotransferase; AMY, amylase;

TBIL, total bilirubin; CHE, cholinesterase; GGT, gamma-glutamyltransferase; CHOL, total cholesterol; TG, triglycerides; HDL, high-density lipoprotein cholesterol; LDL, low-density lipoprotein cholesterol; ALP, alkaline phosphatase; Glu, glucose; HbA_{1c}, glycated hemoglobin; WBC, white blood cells; RBC, red blood cells; Hb, hemoglobin; Hct, hematocrit; Plt, platelets

Supplementary Information

The online version contains supplementary material available at XX.

Supplementary Table 1. Lipemic interference limits for the 27 analytes evaluated in this study

Analyte	Lipemic Interference Limit
TP	Up to 3,000 FTU: No interference
Alb	Up to 3,000 FTU: No interference
Urea	Up to 3,000 FTU: No interference
CREA	Up to 3,000 FTU: No interference
UA	Up to 3,000 FTU: No interference
Na	Up to 1,000 mg/dL: No interferenc
K	Up to 1,000 mg/dL: No interferenc
Cl	Up to 1,000 mg/dL: No interferenc
Ca	Up to 2,000 FTU: No interference
AST	Up to 1,560 FTU: No interference
ALT	Up to 1,560 FTU: No interference
AMY	Up to 3,000 FTU: No interference
TBIL	Up to 5,000 FTU: No interference
CHE	Up to 3,000 FTU: No interference
GGT	Up to 3,000 FTU: No interference
CHOL	Up to 3,000 FTU: No interference
TG	Not applicable*
HDL	Up to 3,000 FTU: No interference
LDL	Up to 3,000 FTU: No interference
ALP	Up to 1,660 FTU: No interference

Glc	Up to 1,410 FTU: No interference
HbA_{1c}	Up to 1,430 FTU: No interference
WBC	Up to 2,579 FTU: No interference
RBC	Up to 2,579 FTU: No interference
Hb	Up to 2,579 FTU: No interference
Hct	Up to 2,579 FTU: No interference
Plt	Up to 2,579 FTU: No interference

Data were obtained from the package inserts and manufacturers of the corresponding reagents and analyzers. LIP refers to the lipemic index that is automatically calculated by the TBA™-2000FR analyzer. Formazin turbidity unit (FTU) or intralipid (mg/dL) values were obtained from manufacturer validation studies. These studies used defined turbidity-inducing substances to evaluate lipemic interference. LIP values are not interchangeable with FTU or intralipid (mg/dL) values. Values represent the highest concentration of lipemia expressed either in FTU or intralipid (mg/dL), at which no significant analytical interference was observed. These thresholds reflect manufacturer-reported data under standardized conditions; the actual interference may vary with the analyzer model, reagent lot, and sample matrix. Thus, they should be interpreted as reference thresholds and not as absolute cut-off values.

*Triglycerides (TG) are the principal lipid components of lipoproteins that cause lipemia; therefore, no lipemic interference limit is applicable to TG, and no FTU threshold has been reported by the manufacturer. All instruments used by the reagent manufacturers for lipemic interference testing are listed below.

iThenticate Page 62 of 71 - Integrity Submission Submission ID trn:oid:::3117:535726404

TP, ALB, CREA, Ca, AST, ALT, TBIL, CHE, GGT, and ALP levels were analyzed using the Hitachi 7180 Clinical Analyzer (Hitachi High-Tech Co., Ltd., Tokyo, Japan). Urea and AMY levels were measured using the LABOSPECT 008 α (Hitachi High-Tech Co., Ltd., Tokyo, Japan). The UA levels were measured using the Hitachi 7170S Clinical Chemistry Analyzer (Hitachi High-Tech Co. Ltd., Tokyo, Japan). Na, K, and Cl were measured using the TBA™-2000FR (Canon Medical Systems Co., Ltd., Otawara, Japan). CHOL, HDL, and LDL levels were measured using LABOSPECT 006 (Hitachi High-Tech Co., Ltd., Tokyo, Japan). Glu was analyzed using the GA09 (A&T Co., Ltd., Yokohama, Japan).

HbA1c was analyzed using the HLC-723® G9 (Tosoh Techno-System Co., Ltd., Tokyo, Japan). The WBC, RBC, Hb, Hct, and Plt were measured using the XE-2100 (Sysmex Co., Ltd., Kobe, Japan).

TP, total protein; Alb, albumin; CREA, creatinine; UA, uric acid; Na, sodium; K, potassium; Cl, chloride; Ca, calcium; AST, aspartate aminotransferase; ALT, alanine aminotransferase; AMY, amylase; TBIL, total bilirubin; CHE, cholinesterase; GGT, gamma-glutamyltransferase; CHOL, total cholesterol; TG, triglycerides; HDL, high-density lipoprotein cholesterol; LDL, low-density lipoprotein cholesterol; ALP, alkaline phosphatase; Glu, glucose; HbA_{1c}, glycated hemoglobin; WBC, white blood cells; RBC, red blood cells; Hb, hemoglobin; Hct, hematocrit; Plt, platelets

Supplementary Table 2. Data and sex-stratified statistical analysis for each analyte in the non-, low-, and high-lipemia groups

Supplementary Table 2-1.

Analyte (unit)	n	Non-lipemia group (n = 54)	Low-lipemia group (n = 157)	High-lipemia group (n = 147)	*P*	*P*		
						Non- vs. low-lipemia group	Non- vs. high-lipemia group	Low- vs. high-lipemia group
CREA (μmol/L)	349	80.89 [67.18–91.72]	79.56 [64.97–96.80]	84.42 [71.60–103.43]	0.161	NA	NA	NA
(mg/dL)		0.92 [0.76–1.04]	0.90 [0.74–1.10]	0.96 [0.81–1.17]				
UA (μmol/L)	248	0.33 [0.29–0.37]	0.35 [0.28–0.41]	0.37 [0.31–0.43]	0.006	0.565	0.014*	0.058
(mg/dL)		5.5 [4.8–6.2]	5.9 [4.7–6.8]	6.3 [5.2–7.2]				
ALT (U/L)	334	22 [16–30]	23 [17–34]	25 [18–42]	0.132	NA	NA	NA
CHE (U/L)	194	296±83	313±100	364±95	<0.001‡	0.718	0.007*	0.002*
GGT (U/L)	318	32 [21–50]	36 [23–73]	42 [27–84]	0.016	0.367	0.016*	0.246
TG (mmol/L)	166	1.42 [1.08–2.18]	4.22 [2.83–5.85]	8.42 [6.38–11.31]	<0.001	<0.001*	<0.001*	<0.001*

(mg/dL)		126 [95–193]	374 [251–518]	746 [565–1001]				
HDL (mmol/L)	129	1.34 [1.09–1.55]	1.14 [0.89–1.38]	0.83 [0.75–1.09]	<0.001	0.071	<0.001*	0.005*
(mg/dL)		52 [42–60]	44 [34–53]	32 [29–42]				
RBC ($\times10^{12}$/L)	331	4.58 [4.12–4.83]	4.50 [3.94–4.89]	4.61 [4.21–4.97]	0.213	NA	NA	NA
($\times10^{6}$/μL)		4.58 [4.12–4.83]	4.50 [3.94–4.89]	4.61 [4.21–4.97]				
Hb (g/L)	331	140 [127–153]	141 [122–152]	146 [133–155]	0.044	1.000	0.476	0.044*
(g/dL)		14.0 [12.7–15.3]	14.1 [12.2–15.2]	14.6 [13.3–15.5]				
Hct (L/L)	331	0.42 [0.39–0.46]	0.42 [0.37–0.45]	0.43 [0.39–0.46]	0.162	NA	NA	NA
(%)		41.9 [39.3–45.7]	41.9 [37.2–44.8]	42.7 [39.4–45.7]				

Supplementary Table 2-2.

Analyte (unit)	n	Non-lipemia group (n = 54)	Low-lipemia group (n = 89)	High-lipemia group (n = 57)	*P*	*P*		
						Non- vs. low-lipemia group	Non- vs. high-lipemia group	Low- vs. high-lipemia group

CREA (μmol/L)	195	62.76 [53.92–72.49]	63.65 [52.16–80.44]	60.11 [50.83–74.26]	0.439	NA	NA	NA
(mg/dL)		0.71 [0.61–0.82]	0.72 [0.59–0.91]	0.68 [0.58–0.84]				
UA (μmol/L)	137	0.27 [0.23–0.34]	0.29 [0.23–0.36]	0.31 [0.26–0.36]	0.430	NA	NA	NA
(mg/dL)		4.6 [3.9–5.7]	4.9 [3.9–6.1]	5.2 [4.3–6.1]				
ALT (U/L)	186	18 [14–26]	23 [15–31]	25 [16–36]	0.162	NA	NA	NA
CHE (U/L)	115	315±83	334±89	379±108	0.017‡	0.621	0.016*	0.090
GGT (U/L)	182	19 [15–34]	27 [18–58]	34 [22–73]	<0.001	0.021*	<0.001*	0.314
TG (mmol/L)	115	1.31 [0.82–1.70]	4.08 [2.42–5.49]	7.03 [4.55–9.82]	<0.001	<0.001*	<0.001*	0.026*
(mg/dL)		116 [73–150]	361 [215–486]	622 [403–869]				
HDL (mmol/L)	84	1.53 [1.21–1.82]	1.33 [1.08–1.99]	1.12 [0.80–1.42]	0.017	1.000	0.018*	0.092
(mg/dL)		59 [47–71]	52 [42–77]	44 [31–55]				
RBC ($\times10^{12}$/L)	180	4.24 [3.97–4.52]	4.22 [3.68–4.45]	4.19 [3.76–4.78]	0.584	NA	NA	NA
($\times10^{6}$/μL)		4.24 [3.97–4.52]	4.22 [3.68–4.45]	4.19 [3.76–4.78]				

iThenticate Page 66 of 71 - Integrity Submission Submission ID trn:oid:::3117:535726404

Hb (g/L)	180	128 [119–135]	129 [109–138]	130 [116–144]	0.342	NA	NA	NA
(g/dL)		12.8 [11.9–13.5]	12.9 [10.9–13.8]	13.0 [11.6–14.4]				
Hct (L/L)	181	0.39 [0.36–0.42]	0.39 [0.34–0.42]	0.39 [0.35–0.43]	0.395	NA	NA	NA
(%)		38.6 [36.4–41.9]	38.7 [34.0–41.5]	39.2 [35.1–43.4]				

Data are presented as median [IQR: 25%–75%] or mean ± standard deviation. Both SI and conventional units are presented. Between-group comparisons were performed using the Kruskal–Wallis test with post hoc Dunn–Bonferroni correction or ‡one-way analysis of variance with post hoc Tukey's test. *$P < 0.05$, considered statistically significant. Sex stratified data are provided separately in Supplementary Table 2-1 (males) and Supplementary Table 2-2 (females).

CREA, creatinine; UA, uric acid; ALT, alanine aminotransferase; CHE, cholinesterase; GGT, gamma-glutamyl transferase; TG, triglycerides; HDL, high-density lipoprotein cholesterol; RBC, red blood cells; Hb, hemoglobin; Hct, hematocrit.

iThenticate Page 67 of 71 - Integrity Submission Submission ID trn:oid:::3117:535726404

Supplementary Table 3. Reference ranges for the 27 analytes evaluated in this study

Analyte (unit)	Reference Range, male	Reference Range, female
TP (g/L)	66–81	66–81
(g/dL)	6.6–8.1	6.6–8.1
Alb (g/L)	41–52	41–52
(g/dL)	4.1–5.2	4.1–5.2
Urea (mmol/L)	2.7–7.1	2.7–7.1
(mg/dL)	8–20	8–20
CREA (µmol/L)	58–94	41–70
(mg/dL)	0.65–1.07	0.46–0.79
UA (µmol/L)	220–463	152–328
(mg/dL)	3.7–7.8	2.6–5.5
Na (mmol/L)	138–145	138–145
K (mmol/L)	3.6–4.8	3.6–4.8
Cl (mmol/L)	101–108	101–108
Ca (mmol/L)	2.18–2.53	2.18–2.53
(mg/dL)	8.8–10.1	8.8–10.1

AST (U/L)	13–30	13–30
ALT (U/L)	10–42	7–23
AMY (U/L)	44–132	44–132
TBIL (μmol/L)	6.8–26.3	6.8–26.3
(mg/dL)	0.4–1.5	0.4–1.5
CHE (U/L)	240–486	201–421
GGT (U/L)	13–64	9–32
CHOL (mmol/L)	3.7–6.4	3.7–6.4
(mg/dL)	142–248	142–248
TG (mmol/L)	0.5–2.6	0.3–1.3
(mg/dL)	40–234	30–117
HDL (mmol/L)	1.0–2.3	1.2–2.7
(mg/dL)	38–90	48–103
LDL (mmol/L)	1.7–4.2	1.7–4.2
(mg/dL)	65–163	65–163
ALP (U/L)	38–113	38–113
Glu (mmol/L)	4.1–6.1	4.1–6.1
(mg/dL)	73–109	73–109

HbA_{1c} (mmol/mol)	30–42	30–42
(%)	4.9–6.0	4.9–6.0
WBC ($\times 10^9$/L)	3.3–8.6	3.3–8.6
($\times 10^3$/μL)	3.3–8.6	3.3–8.6
RBC ($\times 10^{12}$/L)	4.35–5.55	3.86–4.92
($\times 10^6$/μL)	4.35–5.55	3.86–4.92
Hb (g/L)	137–168	116–148
(g/dL)	13.7–16.8	11.6–14.8
Hct (L/L)	0.41–0.50	0.35–0.44
(%)	40.7–50.1	35.1–44.4
Plt ($\times 10^9$/L)	158–348	158–348
($\times 10^3$/μL)	158–348	158–348

This supplementary table presents the reference ranges for the 27 analytes based on the common reference intervals published by the Japanese Committee for Clinical Laboratory Standards Japanese Shared Reference Intervals (2022 edition) [31]. For analytes with sex-specific differences, separate ranges were provided for males and females, whereas for analytes without reported sex-specific differences, identical values were obtained for both sexes.

TP, total protein; Alb, albumin; CREA, creatinine; UA, uric acid; Na, sodium; K, potassium; Cl, chloride; Ca, calcium; AST, aspartate aminotransferase; ALT, alanine aminotransferase;

AMY, amylase; TBIL, total bilirubin; CHE, cholinesterase; GGT, gamma-glutamyltransferase; CHOL, total cholesterol; TG, triglycerides; HDL, high-density lipoprotein cholesterol; LDL, low-density lipoprotein cholesterol; ALP, alkaline phosphatase; Glu, glucose; HbA_{1c}, glycated hemoglobin; WBC, white blood cells; RBC, red blood cells; Hb, hemoglobin; Hct, hematocrit; Plt, platelets

iThenticate Page 71 of 71 - Integrity Submission Submission ID trn:oid:::3117:535726404
