# Supplementary material for: Lipemia and its associations with liver disease and dyslipidemia: a cross-sectional study
Source: Lipids Health Dis. 2025 Dec 27;25:25. doi: 10.1186/s12944-025-02845-7 (PMC12853990; doi:10.1186/s12944-025-02845-7)
Supplement: Supplementary file 5 — Supplementary Material 5 [file 12944_2025_2845_MOESM5_ESM.pdf]

Editing Certificate

This document certifies that the paper listed below has been edited to ensure that the language is clear and free of errors. The logical presentation of ideas and the structure of the paper were also checked during the editing process. The edit was performed by professional editors at Editage, a division of Cactus Communications. The intent of the author's message was not altered in any way during the editing process. The quality of the edit has been guaranteed, with the assumption that our suggested changes have been accepted and have not been further altered without the knowledge of our editors.

MANUSCRIPT TITLE

Lipemia and its Associations with Liver Disease and Dyslipidemia: A Cross-sectional Study

AUTHORS

Mami Osawa

ISSUED ON

September 22, 2025

JOB CODE

QIUXH_2_7

THE EDITAGE Promise QUALITY GUARANTEE

Prabh Grewal
Senior Vice President - Editage

editage helping you get published

Since 2002, Editage has helped over 430,000 authors publish around 1.2 million research papers in scholarly journals across over 1000 disciplines through editorial, translation, transcription, and publication support services. Editage is a brand of Cactus Communications (cactusglobal.com), a science communication and technology company.

CACTUS.

GLOBAL :
+1(669) 272-1214 | request@editage.com

JAPAN :
0120-50-2987 | submissions@editage.com
